# Picomolar concentrations of oligomeric alpha-synuclein sensitizes TLR4 to play an initiating role in Parkinson’s disease pathogenesis

**DOI:** 10.1007/s00401-018-1907-y

**Published:** 2018-09-17

**Authors:** Craig D. Hughes, Minee L. Choi, Mina Ryten, Lee Hopkins, Anna Drews, Juan A. Botía, Maria Iljina, Margarida Rodrigues, Sarah A. Gagliano, Sonia Gandhi, Clare Bryant, David Klenerman

**Affiliations:** 1 Department of Veterinary Medicine, University of Cambridge, Cambridge, CB3 0ES UK; 2 Department of Chemistry, University of Cambridge, Cambridge, CB2 1EW UK; 3 Department of Clinical and Movement Neuroscience, UCL Institute of Neurology, Queen Square, London, WC1N 3BG UK; 4 The Francis Crick Institute, 1 Midland Road, London, NW1 1AT UK; 5 Department of Molecular Neuroscience, University College London Institute of Neurology, London, WC1N 3BG UK; 6 Departamento de Ingeniería de la Información y las Comunicaciones, Universidad de Murcia, 30100 Murcia, Spain; 7 Center for Statistical Genetics, University of Michigan, Ann Arbor, MI USA; 8 UK Dementia Research Institute, University of Cambridge, Cambridge, CB2 0XY UK; 9 Present Address: German Center for Neurodegenerative Diseases, Sigmund-Freud-Straße 27, 53127 Bonn, Germany; 10 Present Address: Weizmann Institute of Science, Perlman Chemical Sciences Building, Room 601, 76100 Rehovot, Israel

**Keywords:** Innate immunity, TLR4, Alpha-synuclein, Aggregation, Parkinson’s disease

## Abstract

Despite the wealth of genomic and transcriptomic data in Parkinson’s disease (PD), the initial molecular events are unknown. Using LD score regression analysis, we show significant enrichment in PD heritability within regulatory sites for LPS-activated monocytes and that TLR4 expression is highest within human substantia nigra, the most affected brain region, suggesting a role for TLR4 inflammatory responses. We then performed extended incubation of cells with physiological concentrations of small alpha-synuclein oligomers observing the development of a TLR4-dependent sensitized inflammatory response with time, including TNF-α production. ROS and cell death in primary neuronal cultures were significantly reduced by TLR4 antagonists revealing that an indirect inflammatory mechanism involving cytokines produced by glial cells makes a major contribution to neuronal death. Prolonged exposure to low levels of alpha-synuclein oligomers sensitizes TLR4 responsiveness in astrocytes and microglial, explaining how they become pro-inflammatory, and may be an early causative event in PD.

**Electronic supplementary material:**

The online version of this article (10.1007/s00401-018-1907-y) contains supplementary material, which is available to authorized users.

## Introduction

Parkinson’s disease (PD) is a common progressive neurodegenerative disease with motor-related symptoms including tremor, bradykinesia and rigidity and characterized by the loss of dopaminergic neurons in the substantia nigra and the presence of Lewy bodies containing deposits of the protein alpha-synuclein (α-syn). Genome-wide association studies (GWAS) have identified several genes including *SNCA,* to be associated with sporadic PD [28] but the molecular causes of the disease and in particular the initial early events that lead to the onset of PD are still largely not understood nor is it understood why the dopaminergic neurons are particularly vulnerable. The aggregation of α-syn, neuroinflammation and oxidative stress all occur during the progression of PD, however, which of these processes occurs first and then initiates the others remains to be established. The temporal order that the initial events occur in matters if one wants to develop treatments for PD that tackle the underlying cause of the disease.

Neuroinflammation in the brain can be protective but there is evidence that with prolonged activation it becomes destructive, playing an important role in the development of neurodegeneration in general and PD in particular [49]. Neuroinflammation is characterised by activation of microglial cells and astrocytes resulting in increased production of cytokines and other pro-inflammatory mediators [54] and can be activated by extra-cellular aggregates of α-syn. Pattern recognition receptors (PRR), such as toll-like receptors (TLRs) detect pathogens which induce inflammation in cells such as macrophages and microglia to control infections [4]. TLR4 senses the Gram-negative bacterial outer membrane component lipopolysaccharide (LPS) and TLR2 detects bacterial lipoproteins, however, both receptors also detect endogenous danger-associated molecules such as proteins produced during tissue damage. Activation of TLR4 and TLR2 recruits myeloid differentiation primary response gene 88 (MyD88) to its cytosolic toll/IL-1R (TIR) domain via the adaptor protein MyD88 adaptor-like (Mal). TLR4 also recruits a second signalling adaptor protein, TIR domain-containing adaptor-inducing interferon-beta (TRIF), via the TRIF-related adaptor molecule (TRAM). It is now clear that oligomeric proteins such as α-syn are recognised by TLRs 2 and 4 [6, 8, 13, 15, 25, 42, 54]. TLRs are also upregulated in the brains of patients with PD [12]. However, the role of TLRs in PD is conflicting when mouse models of the disease have been used. In a MPTP inflammatory model of PD, TLR4 knockout mice were protected [37]. In contrast, TLR4 promoted α-syn clearance in a synucleinopathy protein aggregation mouse model [45]. These contrasting results highlight the importance of establishing the initial events that cause PD in humans and the role played by TLRs. The role of TLRs in the development of PD is also currently unclear because the in vitro studies to date have used 1000-fold higher protein aggregate concentrations than those found in the human disease (estimated at 1–10 pM oligomers in CSF [19, 47]), containing large uncharacterized aggregates over short time courses. Yet PD is chronic in nature so it is difficult to extrapolate the results of these experiments to lower pM doses of smaller soluble aggregates, which will be formed initially during aggregation, over longer times. Furthermore, the differences in α-syn oligomer concentrations between individuals with PD and healthy controls is small, less than a factor of two [20]. This means that healthy controls have pM concentrations of α-syn oligomer but do not develop PD and that there is only a small change in oligomer concentration with the development of PD.

We have first used human genetic and transcriptomic data to investigate the importance of TLR2 and TLR4 in PD. We find evidence to suggest that TLR4 signalling plays a causative role and could contribute to the selective vulnerability of dopaminergic neurons through higher expression of TLR4. To understand the role of TLR4 signalling in PD, we then studied the response of macrophage, microglia and astrocytes to picomolar doses of physiological concentrations of small soluble α-syn oligomers over several days. We find that a TLR4-mediated inflammatory response develops with time due to sensitization. Taking together, these results suggest that TLR4 signalling and the resulting neuroinflammation may be one of the earliest event in PD pathogenesis.

## Methods

### Protein preparation

The expression and purification of wild-type α-syn was performed as detailed previously [7]. Bacterial endotoxins were removed using a detoxi-gel endotoxin removing Columns (ThermoFisher scientific) according to manufacturer’s instruction. Levels in endotoxin-free α-syn were recorded as 0.006 ng/ml (stomacher 80 biomaster seward) in α-syn preparations (autoclaved endotoxin-free plastics and endotoxin-free water were used at all stages of preparations). Protein was then aliquoted into separate eppendorfs into filtered PBS (0.02 μM filter, Whatman) flash-frozen, stored at − 80 °C and thawed once before use. Clusterin was obtained as previously described [11, 50].

### TLR4 antagonists

RSLA and TAK-242 were purchased from Invivogen.

### Protein aggregation

α-syn oligomers were prepared by first diluting in DMEM buffer (DMEM + 1% FCS + 2 mM l-glutamine). The aggregation mixture was incubated for 26.5 h at 37 °C with constant shaking of 200 r.p.m (New Brunswick Scientific Innova 43, 25 mm orbital diameter) and centrifuged at 14200 rpm for 10 min to remove any fibrillar pellets. α-syn fibrils were formed by aggregation for 88 h.

Aggregated α-syn was then stored at 4 °C until incubated with cells. Using thiofalvin-T (ThT) assays, oligomers and fibrils were found to remain stable for 1 week after removal from the shaking incubator, however, aggregates were always used within 24 h.

### ThT assay

The time course of the aggregation was monitored using ThT assays. ThT (Sigma-Aldrich) stocks were prepared in DMSO (Sigma-Aldrich) and diluted into pre-filtered PBS (0.02 μm filter, Whatman) to a final concentration of approximately 100 μM. α-syn was added to 1 ml of ThT solutions and binding monitored by exciting the sample at 440 nm and recording the emission fluorescence spectrum from 460 to 560 nm (slit width 5 nm). Measurements were carried out on a Cary Eclipse spectrofluorometer with a Peltier-controlled holder (Varian, Mulgrave, Australia).

### ThT imaging of unlabeled aggregates

ThT imaging utilized a method previously described [20]. Briefly, glass cover-slides (VWR international, 20 × 20 mm) were cleaned using an argon plasma cleaner (PDC-002, Harrick Plasma) for at least 1 h to remove any residual that fluoresces. 50 µl of poly-l-lysine (70,000–150,000 molecular weight, Sigma-Aldrich) was added to the cover slides and incubated for 1 h before being gently washed with filtered PBS. Imaging was performed on a custom-built total internal reflection fluorescence microscope.

### Single-molecule analysis of labelled aggregates

For single-molecule Förster Resonance Energy Transfer (sm-FRET) experiments, α-syn with the alanine to cysteine mutation at position 90 was used (A90C), and was singly labelled with maleimide-modified Alexa Fluor (AF) 488 or 594 dyes (Life technologies) according to previously established protocols [7, 23] This mutation and fluorescent labelling were demonstrated not to significantly affect the aggregation properties of α-syn in our previous sm-FRET studies of α-syn aggregation [7, 23]. In these previous studies, reproducible conversion of initially disordered oligomers of α-syn to more stable and compact forms was observed during the lag phase of the aggregation reaction. This conformational conversion was demonstrated to be associated with an increase of oligomer cytotoxicity. Low FRET efficiency values were observed in initially formed oligomers (thus termed “low FRET”), while stable toxic oligomers had characteristically high FRET efficiency values, and thus were termed “high FRET”. For the FRET aggregation assay, a 1:1 ratio of α-syn labelled with AF488 (α-syn-AF488) and AF594 (α-syn-AF594) was used, and the same incubation conditions as for other α-syn oligomer preparations (70 µM, 37 °C, with agitation). The solutions were withdrawn after 26.5 h, and analyzed both immediately after withdrawal and after 24.5 h of incubation at 37 °C without agitation, using sm-FRET according to previously described protocols [21]. In sm-FRET experiments, the α-syn solutions were irradiated with a focused 488 nm laser beam, as described previously [21]. As the oligomers contained both α-syn-AF488 and α-syn-AF594 molecules, the α-syn-AF488 non-radiatively excites the α-syn-AF594 via FRET process. Simultaneous fluorescence intensity bursts in both channels are observed for oligomers making them distinguishable from the monomeric protein in solution. The fluorescence intensity values of the oligomeric bursts were used to derive FRET efficiency values (Eq. 1) and apparent size (Eq. 2) for each oligomer.The FRET efficiency values were calculated according to:

1 $$E = \frac{{I_{\text{A}} }}{{I_{\text{A}} + \gamma I_{\text{D}} }}$$

where *I*_A_ is the corrected acceptor fluorescence intensity, *I*_D_ is the corrected donor fluorescence intensity and γ is a gamma factor (0.99) specific to the instrument. FRET efficiency values were represented as histograms with a bin-width of 0.05. The oligomer apparent size was estimated using:

2 $${\text{Apparent }}\,{\text{size}} = 2 \times \frac{{I_{\text{D}} + \gamma^{ - 1} I_{\text{A}} }}{{I_{\text{monomer}} }}$$

where *I*_Dmonomer_ was the average intensity of fluorescence bursts in the donor (AF488) channel after exclusion of oligomeric bursts. The total fluorescence intensity from AF488 is normalized by the average AF488 monomer brightness. The factor of two corrects for the 1:1 stoichiometry of AF488 and AF594 fluorophores. Species determined to be greater than 150-mers or occupying multiple consecutive time-bins were removed from the analysis, under the assumption that they were either fibrillar or arising from dust. The expression is valid under the assumption that there is no appreciable quenching in the soluble oligomers. The size distributions are referred to as “Apparent”; serving as estimates owing to the stochastic nature of fluorescence emission and different trajectories oligomers can take through the confocal volume.

To determine the average FRET efficiency value, the FRET histograms of selected time points were fitted to a Gaussian distribution (Origin 7.0), using the following GaussAmp function:

3 $$y = y_{0} + A\exp \left( { - \frac{{\left( {x - x_{\text{c}} } \right)^{2} }}{{2w^{2} }}} \right)$$

where *A* is the amplitude, *x*_c_ is the centre, corresponding to average FRET efficiency value (E), and *w* the widths of the distribution.

### ELISA assays

To determine cumulative TNF-α, Il-1β and nitric oxide production, supernatants were obtained after incubation with the α-syn over viable time frames and stored at − 80° until analyzed. TNF-α and Il-1β were analyzed using the Duoset^®^ enzyme-linked immunosorbent assay (ELISA) development system (R&D Systems, Abingdon, Oxfordshire, UK). iNOS activity was determined indirectly by measuring using the Griess reaction [18].

### Cell culture

The BV2 cell lines were derived from immortalized murine neonatal microglia. They were grown in Dulbecco’s Modified Eagle’s Medium (DMEM) supplemented with 10% foetal bovine serum and 1% l-Glutamine (Life Technologies) and incubated at 37  °C in a humidified atmosphere of 5% CO_2_ and 95% air, until approximately 1.6 × 10^6^ cell/ml. MyD88^−/−^, TLR2^−/−^ and TLR4^−/−^ murine cells [22, 24, 46] were immortalised previously (a generous gift from Dr D Golenbock, UMass Medical School, USA) and grown from frozen stock samples under the same conditions as BV2. All mice are the same genetic background (C57Bl6).

Astrocytes were from a rat whole-brain mixed glial preparation and prepared following a published protocol that obtains preparations with less than 2% microglia [44]. The brains used were from postnatal Sprague–Dawley (SD) pups. After removing the meninges, the brains were pushed through a 40 µm filter (falcon) using Dulbecco’s Modified Eagle’s (DMEM) Medium (Invitrogen). All filtered brains were collected and homogenized in a falcon tube and spun at 1500 rpm for 5 min. The pellet was resuspended in DMEM media supplemented with 10% fetal bovine serum, 1% penicillin and streptomycin and 1% l-glutamine (Life Technologies) (15 mls per head). Cells were plated out in 25 mL medium per uncoated T75 flask and placed at 37 °C, 5% CO_2_ and left for 5 days to give the glia time to stick down and begin to proliferate. After 5 days, all medium were removed and replaced with 15 ml of fresh medium. Cells were cultured for 14 days before used in experiments and for no longer than 6 weeks after that time. Cells were passaged 2–3 times a week at a confluence of 70–90% after the initial 5 days. Experiments were performed with 1–1.5 million cells per well. To keep the number of microglial very low, we changed the medium every 2–3 days and used uncoated flasks. The cells were sub-cultured 2–3 times per week. For long duration experiments, the buffer was exchanged every 24 h.

### Rodent neuron and astrocyte culture

Cultures of cortical neurons and the co-culture of neuron and astrocyte were prepared from embryos (E17) and postnatal pups of Sprague–Dawley (UCL breeding colony), respectively. Cortices of the brain were placed in ice-cold dissecting buffer (Ca^2+^, Mg^2+^-free HBSS supplemented with 20% fetal bovine serum, sodium bicarbonate and 5 mM HEPES) and washed with washing buffer (dissecting buffer without fetal bovine serum). The tissue was digested with EDTA trypsin for 15 min and neutralized with dissecting buffer (washing buffer supplemented with 400 KU DNAse). Pellets were collected in complete neurobasal medium (NeurobasalA medium supplemented with B28, 2 mM Glutamax and 50 I.U/ml Penicillin/50 ug/ml Streptomycin). Approximately, 50,000 cells for 96-well plates and 100,000 cells for u-slide 8 well ibidi chamber (Thistle Scientific Ltd, Cat no: IB-80826) were plated. Plates were pre-coated with PDL at 37 °C (5% CO_2_) for 2 h. Cells were used at 12–16 days in vitro. Cortical astrocytes were purchased from Caltag Med Systems (Science Cell, Cat no: R1800) which were derived from postnatal day 2 rat cortex. Cells were thawed onto pre-coated plates with PDL (2 h) at 37 °C (5% CO_2_) containing rodent astrocyte medium (Caltag Medsystem, Cat no: 1831) and cultured until full confluence. 100,000 cells were re-plated onto u-slide 8-well ibidi chambers and 50,000 cells were plated onto 96-well plates and used within 2–3 days.

### Human cortical neuron and astrocyte culture

Human cortical neurons (Cat no: 1520) and astrocytes (Cat no: SC-1800) were purchased from Caltag Medsystems Ltd (for Sciencell). Neurons were thawed and plated onto PDL (2 h) and laminin (2 h) pre-coated plates at 37 °C (5% CO_2_) containing neuron medium (Caltag Medsystem, Cat no: 1521) and cultured for 4–5 days until use. Astrocytes were thawed and cultured in PDL-coated 75T flask containing human astrocyte medium (Caltag Medsystmes SC-1801). Upon full confluence, approximately 100,000 numbers of cells were re-plated onto a PDL-coated 8-well ibid chamber and 50,000 cells for 96-well plates. For co-culture preparation, astrocytes were plated on top of neurons (1:1) and cultured for another 3 days until use.

### TNF-α measurements in human astrocytes

Human cortical astrocytes were purchased from Caltag Medsystems Ltd (Cat number: SC-1800) and grown in a PDL-coated 75T flask containing Astrocyte medium (Caltag Medsystmes SC-1801). Upon full confluence, approximately 150,000 numbers of cells were re-plated onto a PDL-coated 8-well ibid chamber (Cat number: IB-80826, Thistle Scientific Ltd) which requires 250 μl medium per well. On the following day, cells were treated with a range of different concentrations of α-syn oligomers. The medium was collected from day 1, day 2, day 3 and day 4, respectively, after treatment. The collected medium was spin down at 15,000 rpm at 4 °C for 10 min to remove cell debris and the supernatant was transferred into a 1.5 mL Eppendorf tube and then stored at –70 °C until use. Autoclaved endotoxin-free plastics and endotoxin-free water was used at all stages of preparations with an endotoxin reading of 0.008 ng/ml recorded. The human astrocytes produce a response to α-syn oligomers but not monomer or fibrils and this response was detectable at oligomer concentrations as low as 5 nM after 72 h of incubation (Supplementary Fig. 7). To study how TLR4 inhibitors affect the release of oligomer-induced TNF-α, human cortical astrocytes were prepared as above. 1 μM TAK242 or 0.1 μg/ml RSLA was pre-treated in cells prior to applying α-syn oligomers. 100 nM Clusterin was co-incubated with oligomer and then applied to the cells. Cells were incubated with each inhibitor for 24 h and then medium was collected as above (Supplementary Fig. 8).

### ROS measurement

ROS production (mainly superoxide) was measured using dihydroethidium (Molecular Probe, 2 μM) as previously described [7]. To inhibit TNF-α activity, either RSLA or TAK242 were pre-incubated in the cells for 30 min prior to imaging and the inhibitors were present during the recording. Clusterin was co-incubated with oligomers for 30 min and then applied together for imaging. 1 μM α-syn monomer or an α-syn oligomeric solution containing 1 μM monomer and approximately 10 nM oligomers with or without a range of inhibitors (RSLA, Clusterin, TAK242) were applied after obtaining basal ROS. Data was analyzed using software from Andor II (Belfast, UK).

### Cell death assay

Cell death was detected using either Sytox green (Molecular Probes) or propidium iodide (PI, Molecular Probes). Cells were washed once and incubated with either 0.5 μM Sytox green or PI and 10 μM Hoechst, nucleic marker (Molecular Probes) in HEPES balanced HBSS (pH adjusted at 7.4 with NaOH) for 15 min. Low-throughput measurements were performed using a Zeiss LSM 710 with a 20× objective and the fluorescence signals were imaged by excitation (ex) 504 nm/emission (em) 523 nm for sytox green. For Hoechst staining, nuclei were imaged using ex 361 nm/em 497 nm to determine the total number of cells. The percentage cell death was quantified by the ratio between the number of green fluorescent and the total number of blue fluorescenct cells per image. 3–5 fields of images were taken per well and data were analyzed using Volocity 6.3, cellular imaging and analysis software. High-throughput images were acquired using a Cellomics Insight NXT. Sytox green staining was imaged by green channel LED and Dapi channel LED was for the hoechst-stained nuclei (17–22 fields of images were taken per wells). The number of fluorescence-positive cells were determined using multi-wavelength cell-scoring module of HCS Studio™ Cell Analysis Software and each experiment kept the same setting of the thresholds (e.g., intensity and size).

### Statistical analysis

A sample size between 2 and 5 individual experiments per conditions was deemed sufficient due to the low variation between replicates. All *p* values for cell experiments where TNF-α production was measured are derived from a one-way ANOVA followed by the Tukey’s post hoc test. For live cell imaging (ROS measurement and cell death), there were total two (human) and three (rodent) independent experiments which include different animals, different cell batches, cell preparation and live-cell imaging. Each set of independent experiment consists of 2–3 wells per condition (for technical replicates) and the figures represent data pooled from all independent experiments. One-way ANOVA with a Tukey correction was used to test statistical significance.

### Gene co-expression network analyses of human brain transcriptomic data

We generated a Gene Co-expression Network (GCN) per brain area using expression data generated by the UK Brain Expression Consortium [40] and assayed with the Affymetrix Exon 1.0 ST Array in the following tissues: cerebellar cortex, frontal cortex, hippocampus, inferior olivary nucleus, occipital cortex, putamen, substantia nigra, temporal cortex, thalamus and intralobular white matter originating from neuropathologically normal individuals. In total, 19,152 microarray probesets were used by the weighted gene co-expression network analysis (WGCNA) R package [26, 27]. In brief, after outlier removal, a “signed” GCN was constructed by creating a signed Topological Overlap Measure (TOM) matrix based on Pearson’s correlation. Gene modules were created by hierarchical clustering based on a 1-TOM dissimilarity matrix. The results of the initial hierarchical clustering were post-processed using the k-means clustering search method with 30 iterations [2]. After this, gene modules were functionally annotated with gProfileR4 R package using Gene Ontology (GO) database without Electronic Inferred Annotations (EIA) and accounting for multiple testing with gSCS.

### GWAS enrichment analysis on co-expression modules

To assess the enrichment of a given GWAS study at modules of a GCN, we used the GWAS summary statistics with VEGAS [29] to perform gene-based tests for association of SNPs and genes. We used HapMap CEU (Central Europeans, Utah) population of reference to estimate patterns of linkage disequilibrium and generate a list of genes and their corresponding *p* value for significance of association with 10,000 simulations and top 10 SNPs for each gene. We use list of pairs (gene, *p* value) in a randomization process for each module of a GCN. Given the set of n genes in the GCN, G, and for a module m, with size k, the genes in that module, G(m), we calculated a observed *z*-score for that module given by

$$z = \frac{{(x - \bar{X})}}{\sigma (X)}$$

where $x = 1/k\mathop \sum \nolimits_{i = 1}^{k} ( - \log 10(pval(g_{i} ))),$ with *g*_*i*_ being the *i*th gene in G(m), $\bar{X}$ corresponds to *x* when applied to all genes in the network, $\sigma (X)$ is the standard deviation of the same values. To obtain an empirical *p* value of the significance of the observed *z*, we generate 10,000 random gene modules, sampling from G, with size k to generate a null distribution of z-scores as in the expression above. Final *p* values are corrected a la Bonferroni for multiple testing. The data flow in Supplementary Fig. 6b shows a visual description of the pipeline.

### Stratified LD score regression analysis

We applied stratified LD score regression [16] to test whether immune-cell-derived genome-wide annotations (eQTLs) were enriched for GWAS heritability of PD [32]. Stratified LD score regression uses GWAS summary statistics to estimate genetic heritability attributed to a particular set of SNPs (i.e., SNPs that are located within genomic regions with the annotation). The method provides a measure of whether this partitioned heritability is significantly enriched in the SNPs harnessing the annotation. We added the annotations individually to the baseline model that has been specified in Finucane et al. [16], and used the HapMap Project Phase 3 SNPs for the regression and the 1000 Genomes Project Phase 3 European population SNPs for LD information. We only considered SNPs with a minor allele frequency > 5%, and we excluded the MHC region from the analysis.

## Results

### TLR4-MyD88 expression and significance in PD-relevant brain regions

We first explored the relevance of TLR4/TLR2-MyD88 signalling in human brain using publicly available cell-specific transcriptomic data [56]. RNA sequencing of purified cell types derived from human cortex demonstrated that while TLR2 is expressed almost exclusively in microglia, TLR4 and MyD88 are expressed by human astrocytes and microglia. Furthermore, comparison with murine cortex showed that TLR4 expression in human glia is higher than in mouse (Supplementary Fig. 1) [55] and that while TLR4 is predominantly expressed by mouse microglia, in humans mature astrocytes also express high levels of this gene [55].

**Fig. 1:**
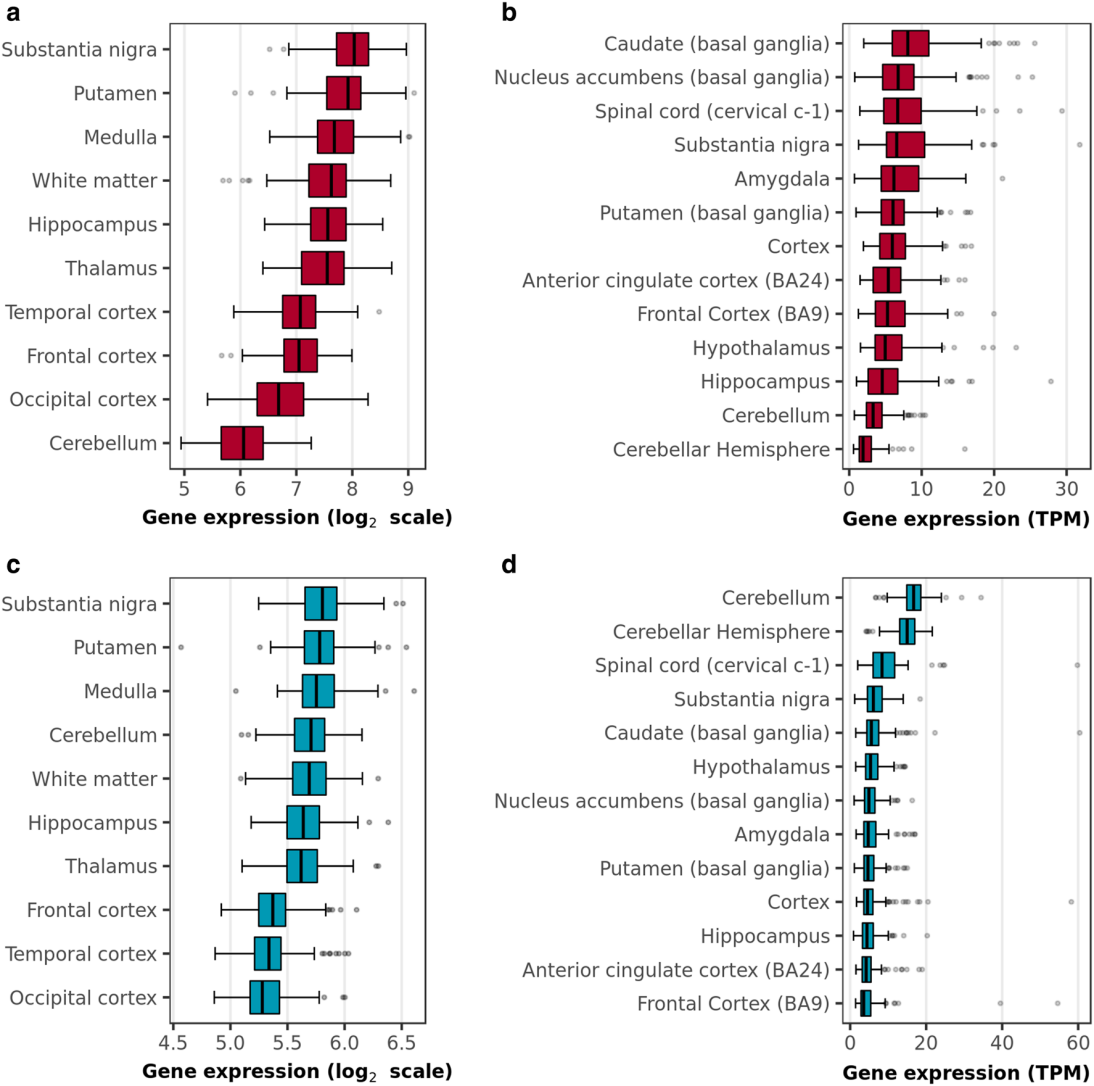
Expression patterns of TLR4 and MyD88 genes in pathologically confirmed normal human brains. **a** TLR4 expression in control human brain as reported by UKBEC. **b** TLR4 expression in control human brain as reported by the GTEx consortium. **c** MyD88 expression in control human brain as reported by UKBEC. **d** MyD88 expression in control human brain as reported by the GTEx consortium. *CRBL* cerebellum, *FCTX* frontal cortex, *HIPP* hippocampus, *MEDU* medulla, *OCTX* occipital cortex, *PUTM* putamen, *SNIG* substantia nigra, *TCTX* temporal cortex, *THAL* thalamus, *WHMT* white matter

Given that cell type-specific RNA sequencing data in humans was derived from only one brain region (temporal cortex), we used publicly available data derived from post-mortem control human brain to further explore TLR4, TLR2 and MyD88 expression across the human brain, including within the substantia nigra and putamen. TLR4 and MyD88 expression was detectable across the human brain using data provided by the UK Brain Expression Consortium (UKBEC, [41] and Genotype-Tissue Expression Consortium (GTEx; [30]) (Fig. 1), which use independent sample sets and different platforms for measuring gene expression. The substantia nigra and putamen were amongst the brain regions expressing the highest levels of these genes according to both resources, though UKBEC data did not include information on the spinal cord. However, we noted some discrepancy in the pattern of MyD88 expression in particular between the UKBEC and GTEx resources, with GTEx reporting MyD88 expression as highest in the cerebellum, whereas UKBEC reported highest expression in substantia nigra. This discrepancy may be due to differences in the platforms used to measure expression or in the brain donation and collection procedures used by the brain banks from which samples were derived [30]. In contrast, TLR2 expression was at the detection limits of microarrays [41] and had very low expression when measured using RNA sequencing [30].

Given the high expression of TLR4 and MyD88 in the substantia nigra, we investigated possible functional relationships between these genes and genes causally implicated in PD through genome-wide association studies (GWAS). Since the vast majority of risk loci for PD are non-coding with the potential to be associated to multiple genes, we used the versatile gene-based association study method (VEGAS) [29] to assign risk SNPs to genes, basing the analysis on GWAS data from the International Parkinson’s Disease Genomics Consortium [32]. This approach incorporates information from all SNPs within a gene and accounts for linkage disequilibrium between SNPs using simulations from the multivariate normal distribution to produce gene-based (as opposed to SNP-based) association p values. Next, we applied gene co-expression analysis to transcriptomic data from control human brain, originating from UKBEC (see Supplementary Fig. 2 for a visual description of the pipeline) with the aim of grouping genes into biologically meaningful modules in an unsupervised manner. In this way, we hoped to identify novel gene relationships, including possible relationships between TLR4-MyD88 signalling and genes associated with complex PD. Finally, we tested all gene co-expression modules for evidence of enrichment of genes associated with PD (as determined using VEGAS) and results showed a significant enrichment within MyD88-containing modules in the putamen and medulla (putamen, enrichment *p* value = 2.20 × 10^−3^; medulla, enrichment *p* value = 2.92 × 10^−2^; Supplementary Table 1).

Interestingly, these modules were not only significantly enriched for PD-associated genes, but for microglial markers (putamen module *p* value = 3.84 × 10^−9^; medulla module *p* value 3.05 × 10^−7^) with some evidence for the more specific involvement of activated (Type 2) microglia in putamen (putamen module *p* value 1.01 × 10^−4^). To robustly asses the genetic evidence for the specific involvement of type 2 microglia in PD, we used stratified LD score regression to determine if functional marks for activated innate immune cells were enriched for genetic heritability. Given the absence of state-specific data for human microglia, we used expression-quantitative trait loci (eQTLs) generated from monocytes at rest and following activation to address this question [14, 39]. This approach demonstrated significant heritability enrichment for PD in eQTLs identified in lipopolysaccharide (Bonferroni-corrected *p* value = 1.05 × 10^−2^), but not interferon-γ-activated monocytes (Bonferroni-corrected *p* value = 0.20) or untreated monocytes (using data from Raj et al. Bonferroni-corrected *p* value = 0.39; using data from Fairfax et al. *p* value = 0.21). Given that monocyte activation by lipopolysaccharide occurs through TLR4-MyD88 signalling these findings suggested that TLR4-mediated activation of microglia/astrocytes generated a cell state of key importance in the development of PD. This finding is consistent with previous reports highlighting the involvement of the immune system in sporadic PD [5, 17, 38, 51].

### Characterization of the oligomeric α-syn used in cell experiments

To determine the mechanism by which physiological concentrations of α-syn oligomers activate microglial and astrocytes, we performed experiments on cells in culture so that experiments could be performed under controlled conditions, using well-characterized solutions of small soluble oligomers. We generated synthetic aggregates of unlabeled α-syn by taking aliquots of α-syn solution directly from an aggregation reaction at the end of the lag phase. Using single-aggregate imaging with Thioflavin T (ThT), we counted the number of aggregates present to ensure that each reaction was reproducible [20].

Our previous work using single-molecule Förster Resonance Energy Transfer (sm-FRET) aggregating the same concentration of dye-labeled α-syn monomer showed that approximately 1% of the total solution would be oligomeric [7, 23]. We have confirmed these sm-FRET results using both SEC [6] and TEM [20]. The small-soluble high-FRET oligomers that are formed under these conditions, by a slow conversion process from low-FRET oligomers, were found to be the most toxic species producing increased ROS when added to a neuron and astrocyte co-culture [7, 23]. Once formed, the oligomers are stable for several hours enabling us to dilute the stock aggregation mixture to produce solutions of known initial oligomer and monomer concentration. However, since aggregation was performed in a slightly different buffer to be compatible with cell culture, we repeated the oligomer characterization.

Experiments using sm-FRET [7, 23] showed that the % of oligomers in our preparation was 1.5 ± 0.5% (*n* = 3, SD) at 26.5 h of incubation and 1.7 ± 0.4% (*n* = 3, SD) after 50 h (26.5 h of incubation with shaking followed by 24.5 h of static incubation) see Supplementary Fig. 3. The derived FRET efficiency histograms were centered at *E* = 0.65 (Eq. 3), corresponding to high-FRET oligomer type, also in good agreement with previous measurements [7, 21, 23] and confirmed that they were smaller than 20 mers.

It is also possible that additional oligomers are formed during the incubation with cells for 24 h before buffer is exchanged. To test this, we performed experiments with unlabeled protein. Both monomer only and preformed oligomers were added to cells (Supplementary Fig. 4). α-syn monomer was only incubated with BV2 microglia cells at concentrations ranging between 10 pM–4000 nM and left for 24–120 h. The supernatant of the cells was then removed and analyzed by either ThT fluorescence or ELISA. Under conditions where α-syn monomers were incubated with the microglia cells at a concentration less than 1 nM, no significant (*p* = 0.8) increase in fluorescence was observed using bulk ThT assays and no increase in cytokine production was recorded with ELISA’s. 200 nM monomer left for 24 to 48 h also showed very minimal aggregation but oligomer formation was detectable for longer times, since an increase in TNF-α production and ThT signal was observed and aggregates could be detected by direct imaging. Similarly, 1000 nM monomer left for 24 h showed minimal aggregation but significant TNF-α production beyond this time compared to the media control (*p* = 0.006). We also added preformed oligomers, with monomer, to cells. Direct imaging of the number of aggregates in the solution before and after incubation with cells for 24 h, using ThT imaging, confirmed that there was only a detectable increase in oligomer number at 1000 nM monomer (approximately double) and no detectable increase at lower monomer concentrations. Overall, these experiments show that for incubations below 1000 nM monomer and 15 nM oligomers, there is no significant increase in oligomer concentration over the 24-h incubation.

To determine how long we could perform experiments, cell survivability experiments were performed. Cells were grown to a density of 0.4 × 10^6^ cells/ml and α-syn oligomers in new cell media introduced. Supernatants were taken each day and fresh buffer added, after wells were washed in LPS-free PBS. Four concentrations of oligomeric α-syn were used to test the effect oligomer concentration had on survival. An approximate 90% survival (compared against hour 0) in cell population was observed up to the 96 h mark with 1000 nM oligomer or lower, see Supplementary Fig. 5.

### Oligomeric α-syn-induced cytokine production is predominantly generated by TLR4-dependent MyD88 signalling

**Fig. 2:**
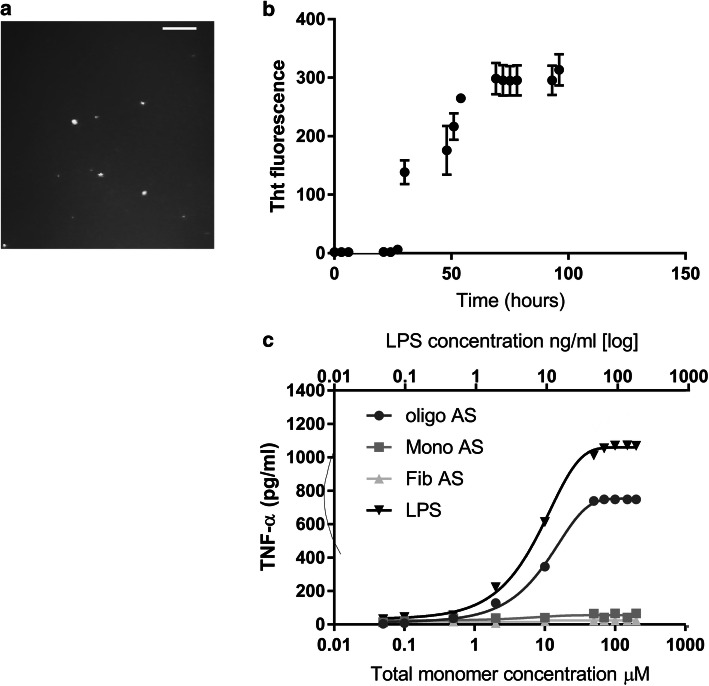
Aggregation of αS and the pro-inflammatory response of microglial cells to different αS aggregates. **a** ThT single-molecule fluorescence imaging of α-syn aggregates after 24 h of aggregation of 10 µM monomer. Significant increase of aggregates (*p* < 0.01) is observed. Scale bar represents 5 µm. **b** Bulk  ThT assay of α-syn  (10 µM)  aggregation when left at 37 °C in a shaking incubator (200 rpm) over a period of 96 h (*n* = 8 sem). **c** The pro-inflammatory response, as measured by TNF-α production, of BV2 microglia after a 24-h incubation with αS-only monomers, oligomers, and fibril samples (0.05–200 μM total monomer) compared to LPS (0.05–200 ng/ml) stimulation (*n* = 5, sem). Monomer or fibrils produce significantly less TNF-α compared to oligomers over all concentrations (monomers *p* = 0.0029, fibrils *p* = 0.0018, one way ANOVA with post hoc Tukey)

Initial experiments established that soluble oligomers, but not monomers or fibrils, induced an inflammatory response from the same initial preparation of α-syn monomer (Fig. 2). This confirmed that there was minimal or no LPS contamination in the α-syn preparation. Immortalised murine microglia cells (BV2) were incubated with monomeric, oligomeric or fibril forms of α-syn at varying monomer concentrations and left at 37 °C for 24 h. Levels of TNF-α, IL-1β and NO were then measured in the cell supernatant by ELISA (Fig. 2). As expected, the incubation of these cells with the TLR4 agonist LPS (at ng/ml concentrations) induces robust activation of this receptor.

The α-syn oligomers (70 µM total monomer containing approximately 100 nM oligomers) produced 750 pg/ml of TNF-α in comparison to the same concentration of the monomer or fibril solutions (40 and 25 pg/ml, respectively). At this oligomer concentration, considerable cell death occurred by 24 h (33% cell death compared to day 0, see Supplementary Fig. 5a). Increasing concentrations of fibril or monomer samples (Fig. 2c) failed to show a significant increase in TNF-α production confirming that only oligomers cause an inflammatory response, as previously reported [6, 8].

**Fig. 3:**
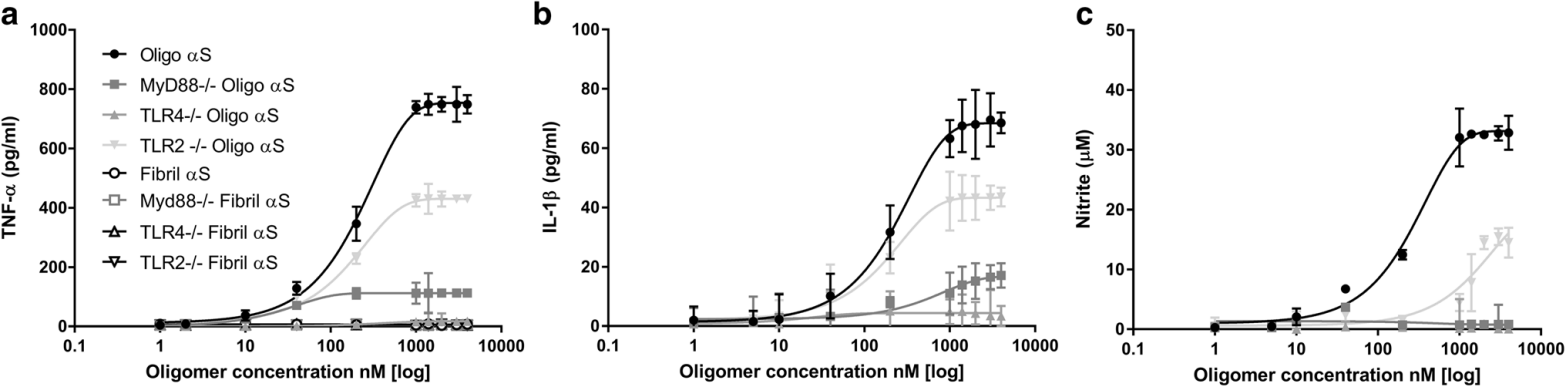
Pro-inflammatory response of TLR4, MyD88 or TLR2 knockout macrophages to αS oligomers. Cells were stimulated with αS oligomers (1–4000 nM (total monomer concentration)) or αS fibrils for 24 h. The levels of the pro-inflammatory mediators TNF-α, IL-1β and NO were measured. **a** TNF-α production is significantly reduced with increasing oligomer concentration in the knockout cells compared to wild-type (TLR4^−/−^ *p* = 0.0015, MyD88^−/−^ *p* = 0.006, TLR2^−/−^ *p* = 0.02) (*n* = 5, sem). **b** IL-1β production is significantly reduced with increasing oligomer concentration in the knockout cells compared to wild-type (TLR4^−/−^  *p* = 0.0015, MyD88^−/−^ *p* = 0.006, TLR2^−/−^ *p* = 0.008) (*n* = 4, sem). **c** NO production is significantly reduced   with increasing oligomer concentrations in the knockout cells compared to wild-type (TLR4^−/−^ *p* = 0.0014, MyD88^−/−^ *p* = 0.002, TLR2^−/−^ *p* = 0.03 (*n* = 3, sem). All statistical comparisons among groups were performed using one-way ANOVA, followed by the Tukey’s post hoc test

To determine which receptors were involved in the immune response, previously characterized immortalized murine bone marrow-derived macrophage cells were used derived from mice lacking either TLR4, TLR2 or MyD88 [22, 24, 46]. These cells were incubated with different concentrations of α-syn oligomers or monomers for 24 h. The cytokine levels present in the cell supernatant were then analyzed by ELISA (Fig. 3). As expected in wild-type cells, cytokine production was seen only with oligomers and not monomers.

In TLR4^−/−^ cells, the inflammatory response to oligomers (TNF-α, Il-1β and NO) was significantly reduced compared to wild-type cells, ~ 10 to ~ 100 fold decrease. Likewise, in MyD88^−/−^ cells and to a much lesser degree in TLR2^−/−^ cells there was statistically significant decreased TNF-α, IL-1β and NO production compared to wild-type cells. Overall, these experiments show that TLR4 signalling through MyD88 is the dominant pathway by which α-syn oligomers are recognised by macrophages. TLR2 plays a more minor role in α-syn-induced inflammation. Since only α-syn oligomers cause signalling, the α-syn oligomer concentration used here is quoted for the rest of the paper.

### Low concentrations of α-syn oligomers sensitizes inflammatory responses through TLR4

**Fig. 4:**
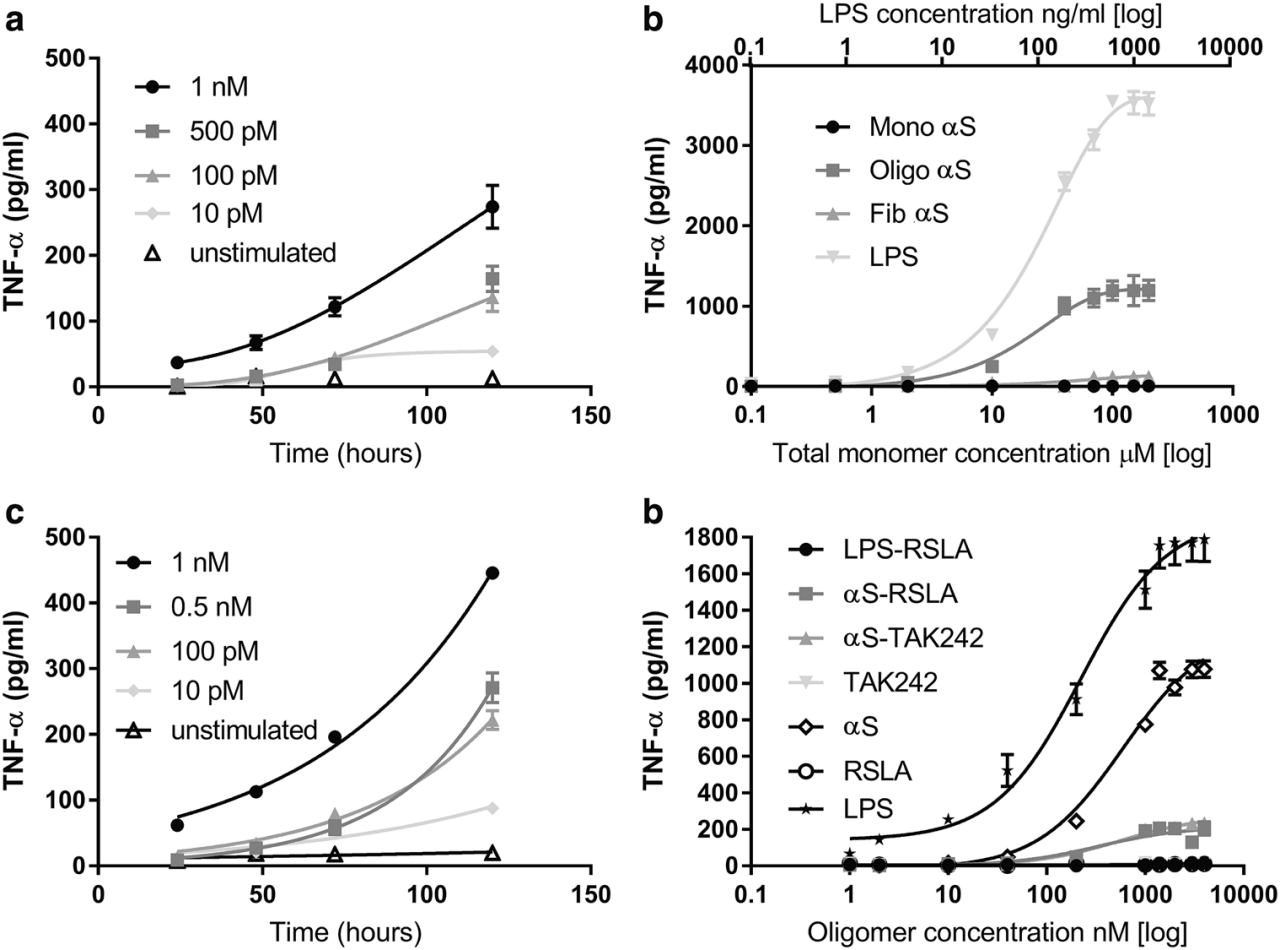
Response of BV2 microglial cells and astrocytes to α-syn oligomers and the blocking of TNF-α production by TLR4 antagonists. **a** Time course of TNF-α production by BV2 cells in response to sustained exposure to αS-oligomers (10 pM–1 nM (*n* = 3, sem). Buffer was exchanged every 24 h. **b** The response of astrocytes to αS-monomers, αS-oligomers and αS-Fibrils (0.05–200 μM (total monomer concentrations)) compared to LPS (0.05–200 ng/ml) stimulation stimulation for 24 h (*n* = 4, sem). A statistically significant increase was observed in response to oligomer vs monomers (*p* = 0.0041) and in oligomer vs fibrils (*p* = 0.0078). **c** Time course of TNF-α production by astrocytes in response to sustained exposure to αS-oligomers (10 pM–1 nM). Buffer was exchanged every 24 h (*n* = 4, sem). After 72 h a significant increase in response was observed in response to αS-oligomers compared to unstimulated cells (*p* = 0.009 at 72 h, *p* = 0.0036 at 120 h). **d** The response of astrocytes after a 24 h incubation with αS-oligomers only, αS-oligomers and the TLR4 antagonist RSLA and TAK242, LPS with RSLA and RSLA only (*n* = 3, sem). Both RSLA (*p* = 0.0165) and TAK-242 (*p* = 0.0204) show a statistically significant decrease when added with αS-oligomers, compared to αS-oligomers alone. All statistical comparisons among groups were performed using one-way ANOVA with post hoc Tukey test

Oligomer concentrations in the CSF of patients suffering from PD are of the order of 1–10 pM. We, therefore, incubated BV2 microglial cells with lower concentrations of α-syn oligomers (1 nM–10 pM), together with α-syn monomer (100–1 nM), for a sustained period of time (up to 5 days; Fig. 4a) and measured supernatant cytokine production at 24, 48, 72 and 120 h. Lower oligomer concentrations no longer killed the BV2 cells. Significant (*p* = 0.009) induction of pro-inflammatory cytokines in response to α-syn oligomers, compared to unstimulated cells, was observed. Rat astrocytes produce a response to α-syn oligomers but not monomer or fibrils (Fig. 4b) and this response was detectable at low pM oligomer concentrations after 72 h of incubation (Fig. 4c, *p* = 0.009, compared against unstimulated cells). There was no significant cytokine production in TLR4 knockout macrophage cells (Supplementary Fig. 6) indicating that TLR4 is required for signalling. By 120 h α-syn oligomer stimulated cells started to die so longer incubations times were not feasible (Supplementary Fig. 5a). This α-syn sensitization response was effectively inhibited by the TLR4 antagonists RSLA or TAK-242 (Fig. 4d).

Assuming increased mRNA expression of TLR4 in human glia is indicative of higher protein expression then this would be expected to result in increased levels of TNF-α on exposure to oligomer concentrations. Experiments on cultured human astrocytes explicitly tested this prediction and confirmed that sensitization also occurred at low-oligomer concentrations (Supplementary Fig. 7) and was significantly reduced by TLR4 antagonists (Supplementary Fig. 8).

### α-syn oligomer-induced inflammatory sensitization is reversible

**Fig. 5:**
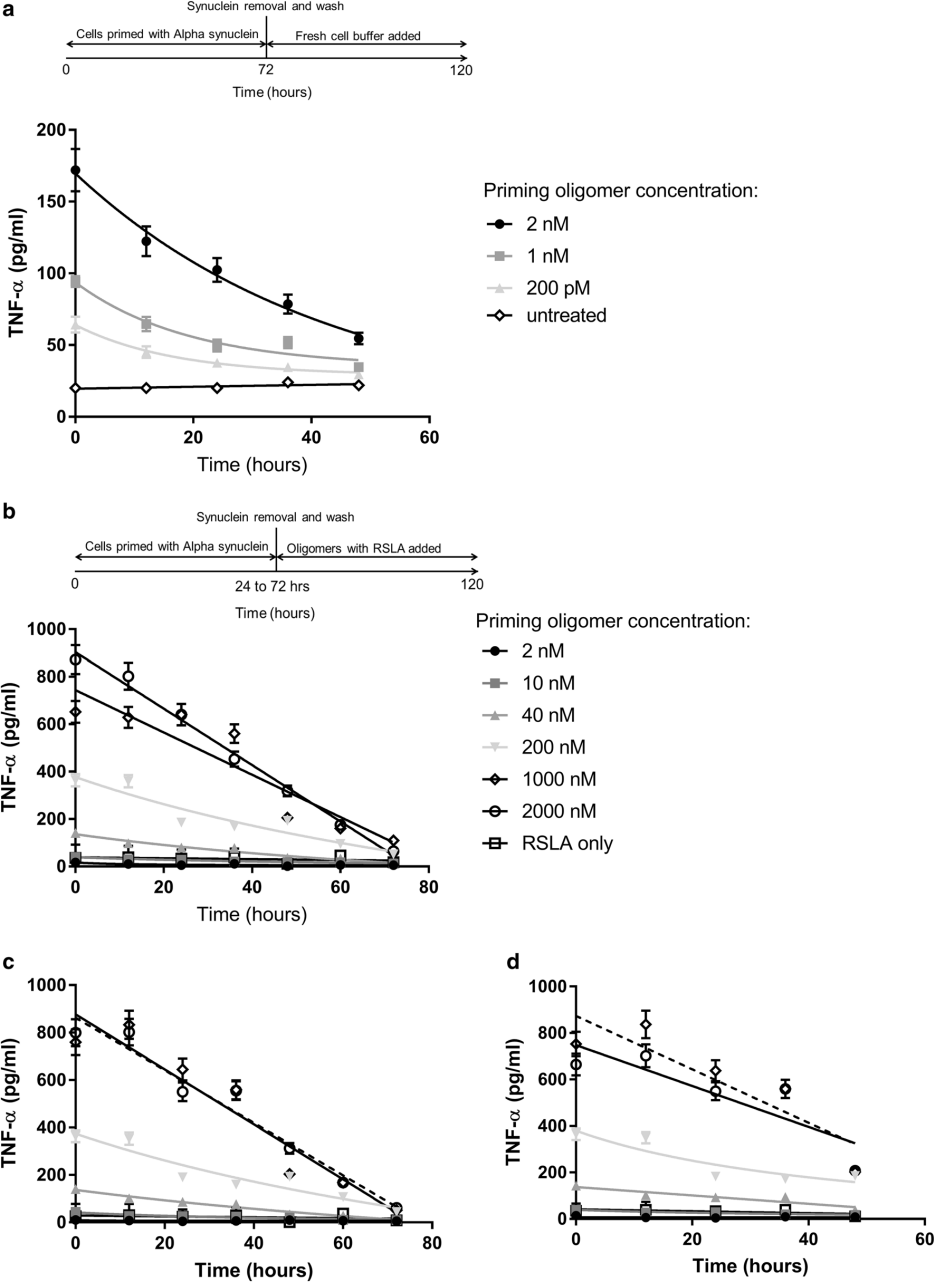
Reversal of α-syn oligomer-induction of cell priming. The pro-inflammatory response (induction of TNF-α) by BV2 microglia after priming with oligomeric α-syn was determined by ELISA. **a** BV2 microglia were primed with 2 nM, 1 nM or 200 pM concentrations of α-syn oligomers for 72 h before α-syn was removed and replaced with fresh buffer. TNF-α levels were measured every 12 h by replacing the solution above the cells with fresh buffer. The production of TNF-α decreases slowly with time, but is not completely restored to the unprimed state (*n* = 3, sem). 2 nM, 1 nM and 200 pM oligomer is significantly higher than untreated cells at all times (*p* = 0029, *p* = 0.0056 and *p* = 0.087 respectively). **b**–**d** BV2 microglia were incubated with oligomeric α-syn, 2–2000 nM. Cells were primed for different lengths of time (**b** 24-h, **c** 48-h, **d** 72-h) before α-syn oligomers were removed, cells were washed with media and replaced with media containing α-syn oligomers and the TLR4 antagonist RSLA. RSLA reduced TNF-α production and enhanced cell survival returning viability to 90% (± 3%) over the duration of the experiment (Supplementary Fig. 5b) (*n* = 4, sem). All statistical comparisons among groups were performed using one-way ANOVA with post hoc Tukey test

To determine whether microglia could recover if α-syn oligomers were removed from the cells we measured TNF-α production over time, after exposure of BV2 microglia to α-syn oligomers for 72 h followed by oligomer removal (Fig. 5). The production of TNF-α decreases slowly with time, but is not completely restored to basal levels after 2 days. To determine whether TLR4 inhibitors could also reverse α-syn oligomer-induced sensitization, BV2 cells were incubated with α-syn oligomers for 24–72 h. Cells were washed and the media replaced with RSLA and fresh α-syn oligomers and a marked reduction in TNF-α production was seen in the following 48–72 h (Fig. 5b–d). Cell viability showed no significant change (Supplementary Fig. 5b). These data suggest the α-syn sensitization is reversible if α-syn oligomers are removed from the cells or if the cells are incubated with a TLR4 antagonist even if α-syn oligomers are still present in the media.

### TLR4 antagonists protect neurons from oligomer-induced toxicity

α-syn oligomers can cause excessive ROS generation, and cell death in neurons. To determine the role of TLR4 signalling in ROS and cell death we used TLR4 antagonists. We also used an extracellular chaperone, clusterin, to bind the oligomers and block all oligomer-induced processes. Clusterin preferentially binds amyloid beta oligomers over monomers [33, 34] and been shown to have an effect on α-syn [43].

**Fig. 6:**
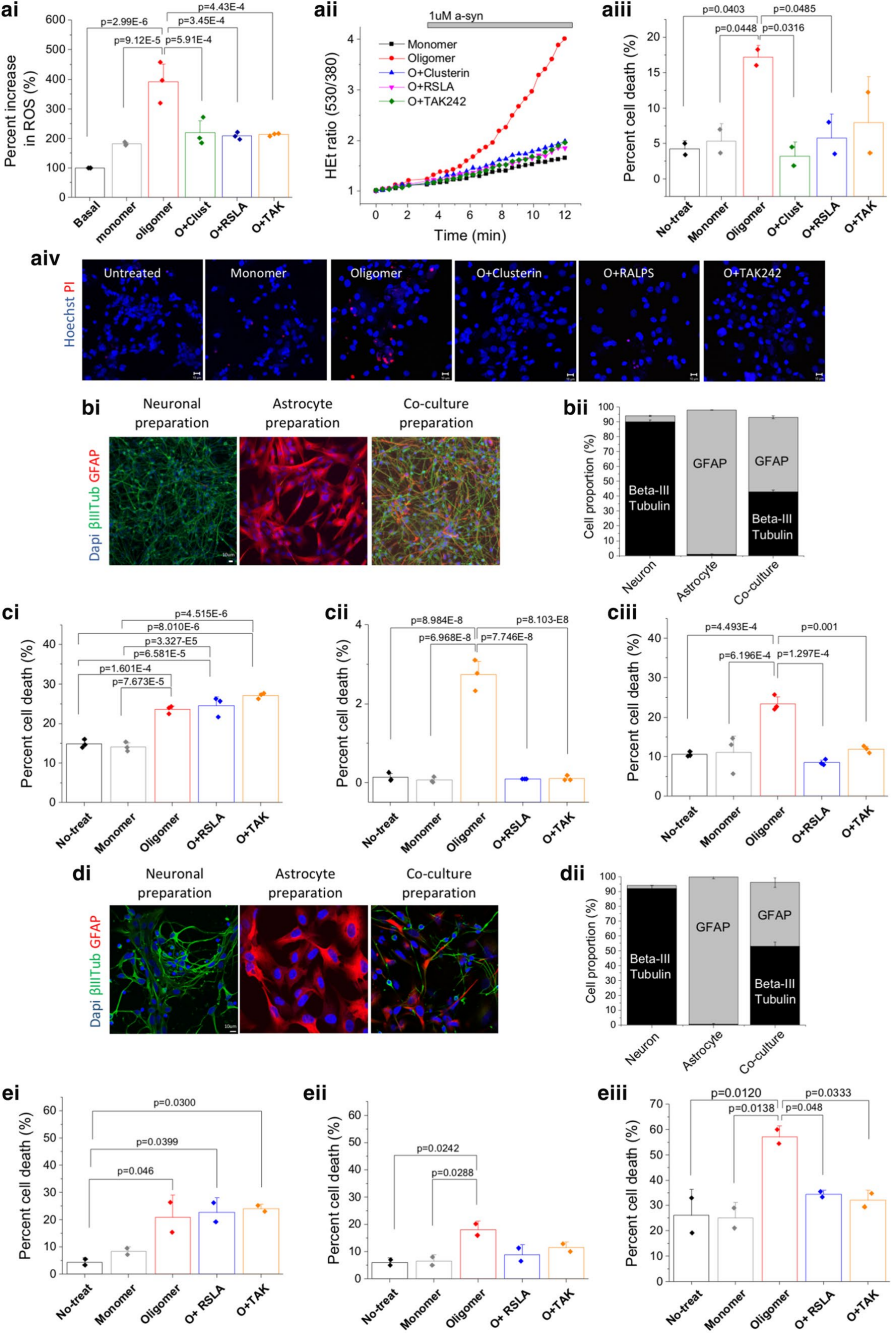
Live cell imaging of α-syn oligomer induced cell toxicity, and dependence on TLR4. An oligomeric solution containing 1 μM monomer and approximately 10 nM oligomers were used with or without a range of inhibitors (RSLA, Clusterin, TAK242). **ai** α-syn oligomer induced ROS was measured in rat cortical neuron and astrocyte co-culture using a ratio of dihydroethydium (Het) fluorescence between its oxidized and non-oxidized forms. 0.1 μg/ml RLSA or 1uM TAK242 reduced overproduction of ROS induced by α -syn oligomer (*n* = 3, sem). Clusterin, RSLA or TAK242 alone did not produce an increase in ROS (data not shown). **aii** Representative traces from the ROS measurement show that treatment with TLR4 inhibitors reduced the ratio of Het fluorescence in comparison to α-syn oligomer alone. **aiii** Cell death assay was performed using Propidium Iodide (PI) under the same conditions but cells were incubated overnight with each inhibitor, Clusterin, RSLA or TAK242 (*n* = 2, sem). Oligomers induced an increase in cell death whilst all three inhibitors prevented cell death. **aiv** Representative images from cell death assay. **bi** Representative images showing rodent neurons and astrocytes **bii** Quantification of cell type in cultures. **ci**–**iii** Cell death was measured in enriched rodent neurons and astrocytes and co-cultures: (**i**) neurons, (**ii**) astrocyte, (**iii**) co-culture preparation. (*n* = 3, sem). **di** Representative images showing human neurons and astrocytes. **dii** Quantification of cell type in cultures. **ei**–**iii** Cell death was measured in enriched human neurons and astrocytes and co-culture: (**i**) neurons, (**ii**) astrocyte, (**iii**) co-culture preparation. (*n* = 2, sem). No-treat: no treatment, Monomer: 1 μM a-syn monomer, Oligomer; 10 nM α-syn oligomer, O + RSLA: 10 nM α-syn oligomer + 0.1ug/ml RSLA, O + TAK: 10 nM α-syn oligomer + 1 μM TAK242. All statistical comparisons among groups were performed using one-way ANOVA, followed by the Tukey’s post hoc test

To test the effect of clusterin, RSLA and TAK242 on the production of ROS, we used dihydroethidium (DHE) dye which allowed us to measure the rate of oxidation of the dye by cellular superoxide production. Rat co-cultures of neurons and astrocytes were treated with either 1 μM TAK242 or 0.1 μg/ml RA-LPS for 30 min prior to application of oligomers to the culture. Clusterin was pre-incubated with α-syn oligomers before application to the cells. The production of ROS (mainly superoxide) after application of the α-syn oligomers was compared to the basal level (Fig. 6a). The results showed that α-syn oligomers produced significantly increased levels of ROS, approximately 4-fold as shown previously, and this overproduction was prevented in cells pre-treated with TLR4 inhibitors, RSLA and TAK242. ROS production was also reduced by pre-incubation with clusterin. Application of oligomers for 24 h induced cell death, as previously shown. Oligomer induced cell death in the rat co-culture was reduced to basal levels by both clusterin and the TLR4 antagonist RSLA (Fig. 6aiii). Interestingly, clusterin was less effective when used in experiments on BV2 cells and significantly higher clusterin concentrations were needed for a reduction in TNF-α to be observed (Supplementary Fig. 9). Clusterin needs to bind to the oligomers to prevent TLR4 signalling but only a few TLR4 receptors need to signal for cytokine production. Therefore, both the number of TLR4 receptors and the number of oligomers that are not bound or partially bound by clusterin will determine the extent of TLR4 signalling and resulting cell death. This result suggest that targeting TLR4 directly may be a more effective strategy than binding the oligomers, especially if TLR4 expression is high as is presumably the case with BV2 cells.

Next, we tested whether TLR4 inhibitors reduced the α-syn oligomer-induced cell toxicity in rodent neurons, astrocytes and co-cultures of neurons and astrocytes (Fig. 6ci–iii, ei–iii). Cell preparations were characterized for neuronal and astrocyte enrichment using the markers β-III Tubulin and GFAP (Fig. 6bi). Neuron-enriched cultures contained 90% ± 1.3% β-III Tubulin-positive cells and astrocyte enriched cultures contained 97% ± 0.1% GFAP-positive cells. Neuron-astrocyte co-cultures contained 43 ± 1.1% neurons and 50 ± 1.2% astrocytes (Fig. 6bii). Cells were incubated with α-syn oligomers with or without RSLA or TAK242 overnight, and cell death was assessed by automated counting of the number of dead cells (labelled with sytox green) and number of total cells (Hoechst) (Supplementary Fig. 10a). We also measured the amount of TNF-α production (Supplementary Fig. 11a). Neuronal cell death was significantly increased by α-syn oligomers (*p* = 7.673E-5) in the enriched neuronal cell culture, and this was not affected by TLR4 blockade (Fig. 6ci). Oligomers also induced very low level, but highly significant, toxicity to astrocytes (*p* = 6.968E − 8), and this was prevented by TLR4 antagonists (RSLA; *p* = 7.746E-8, TAK242; *p* = 8.103E − 8, Fig. 6cii). Notably, the oligomers induced significant cell death in the neuron-astrocyte co-culture (*p* = 4.493E-4), and this was reduced to basal levels by the presence of the TLR4 inhibitors (RSLA; *p* = 1.127E-4, TAK242; *p* = 0.001, Fig. 6ciii) and TNF-α levels reduced to basal levels (Supplementary Fig. 11a). This data suggested that astrocytes are able to drive cell death in rodent co-cultures through oligomer-induced TLR4 signaling.

As we have reported that α-syn oligomers drive human astrocytic production of TNF-α (Supplementary Figs. 7, 8) via TLR4-dependent pathways, we tested the effect of TLR4 inhibition on oligomer induced cell toxicity in a primary human model (Fig. 6d, e). We generated enriched human neuronal cultures (92 ± 2.1% β-III Tubulin-positive cells), enriched human astrocyte cultures (99 ± 1.2% GFAP) and a neuron-astrocyte co-culture (β-III Tubulin: 52 ± 2.8%, GFAP: 43 ± 3.2%, Fig. 6dii). Application of α-syn oligomers induced significant levels of cell death in both the enriched neuron (*p* = 0.046, Fig. 6ei) and the enriched astrocyte (*p* = 0.0288, Fig. 6eii) cultures. As noted with the rodent cells, the oligomer-induced neuronal death was not abolished by TLR4 blockade in the absence of astrocytes (Fig. 6ei). TLR4 antagonism did show a tendency to prevent oligomer-induced astrocytic cell death (Fig. 6eii). Importantly, in the human neuron-astrocyte co-culture experiment, oligomer-induced cell death was prevented by the application of TLR4 antagonists RSLA and TAK242 (*p* = 0.048 and *p* = 0.0333, respectively, Fig. 6iii, Supplementary Fig. 10b) and the TNF-α levels reduced to basal levels (Supplementary Fig. 11b).

We estimated the proportion of astrocytic and neuronal cell death in the co-culture experiment, by sampling the nuclear size of neurons versus astrocytes from the enriched cultures, and segmenting the sytox green-positive nuclei on the basis of size in the co-cultures (Supplementary Fig. 12). For both the rodent and human co-culture, the majority of the oligomer-induced cell death was neuronal rather than astrocytic (91 ± 2.6% in rodent and 68 ± 2.9% in human co-culture preparation).

Taken together, this data shows that oligomers are able to induce neuronal toxicity through two mechanisms: neurons alone are directly susceptible to oligomer-induced TLR4-independent toxicity. However, in the presence of astrocytes, the dominant mechanism of oligomer-induced neuronal cell death is TLR4-dependent oligomer-induced activation of astrocytes leading to the production of TNF-α and other cytokines by astrocytes.

## Discussion

Sporadic PD is not caused by a single-gene mutation but rather a large number of different genes and environmental factors contribute. For this reason, we used transcriptomic data from humans as a starting point to determine the relevance of TLR2 and TLR4 signalling in the initiation of PD. Our analysis of human brain transcriptomic data shows that of all brain regions analyzed, the substantia nigra has the highest levels of TLR4 expression. Furthermore, integrative analyses, which make use of PD GWAS data with gene co-expression networks in human brain, suggest a causal role for microglial MyD88-dependent signalling in PD. Taken together, this supports the concept that TLR4-MyD88 signalling via both astrocytes and microglial plays a key role in the onset of PD and explains the selective vulnerability of the substantia nigra. To elucidate the mechanism by which this occurred, we then performed experiments at physiological concentrations of oligomers. Under these conditions, α-syn oligomers are capable of provoking an immunological response from microglia and astrocytes and TLR4 receptors produce the majority of the activation of cytokine production. Immortalised mouse microglial cells as well as TLR4 and MyD88 knockout macrophage cells have all been used to test the cytokine response to α-syn oligomers. When MyD88 is absent, the cells were still able to produce a low level of inflammatory cytokines suggesting α-syn oligomer-induced TLR4-dependent cytokine production is also being induced by TRIF-dependent signalling [52]. Our work suggests that TLR2 plays a minor role in both the α-syn-induced TNF-α production and the initiation of PD. This is in agreement with other published work [15, 42] although some studies propose a more dominant role for this receptor [8, 25]. TLR2 expression is upregulated upon activation of TLR4 [48] so it is possible that α-syn activation of TLR4 not only leads to direct generation of inflammatory signalling, but also enhances inflammatory signalling through other TLRs. The increase in expression of TLR2, for example, may either result in enhanced sensitivity of this receptor to TLR2 ligands and/or to a net increase in TLR2 signalling to these ligands and occur later in the progression of the disease. Importantly, our data shows a significantly higher sensitivity of TLR4 to oligomers than TLR2 at low oligomer concentrations suggesting that TLR4 will be the major receptor at physiological conditions and that TLR4 signalling involves MyD88.

Our data show that there is a sensitization or training [35] of the immune response in macrophage and microglial responsiveness over time to a constant low-level dose of α-syn oligomers. Sensitization or priming has been reported for experiments using LPS, which acts via TLR4, as a systemic challenge [31] but not by protein aggregates, to our knowledge and not by continual dosing. Low levels of LPS lead to an increased reaction to a secondary challenge [9], although the mechanism by which this occurs remains to be established. In contrast, high doses of LPS lead to tolerance [53]. Furthermore, it is known that cells become sensitized with age [1] and also, once primed, give a larger response to a secondary challenge such as addition of LPS or infection [36]. Importantly, and in contrast to the LPS effects, we observed sensitization to continual low-dose exposure. Therefore, no increase in oligomer concentration, and no rechallenge experiment is required to demonstrate the effect, allowing us to utilize a physiologically relevant experimental paradigm.

While it is well known that protein aggregation, neuroinflammation and oxidative stress all contribute to the onset of PD, it is not known what the initial cause is. Our experiments show that an inflammatory response can occur without any increase in oligomer concentration from the physiological levels. The production of cytokines such as TNF-α leads to oxidative stress which is known to lead to altered mitochondrial function [10]. These conditions can then result in increased protein aggregation, further increasing TLR4 signalling, and leading ultimately to the formation of Lewy Bodies and cell death. While there are potential issues extrapolating from these cell experiments to animals, this finding is also supported by the MPTP inflammatory model of PD, where TLR4 knockout mice were protected [37]. Prolonged exposure to low levels of TLR4 agonists leads to switching to a pro-inflammatory response, which increases with time and so cannot be reversed without intervention. Furthermore, it is recognized that patients with PD suffering from intercurrent infective/inflammatory illnesses undergo deterioration in their PD, and a variety of reasons have been suggested to underlie this [3]. Our data would support the hypothesis that systemic inflammatory cytokines may accelerate the cytokine-induced neuronal damage that drives PD pathogenesis.

It has been extensively reported that oligomers interact directly with neurons to induce synaptic dysfunction, calcium dysregulation, mitochondrial dysfunction and oxidative stress. Here, we show that significant neuronal damage is mediated by the interaction between α-syn oligomers and astrocytes/microglia resulting in the release of cytokines that cause damaging effects on neurons. Our transcriptomic and genomic analysis shows that this interaction appears crucial in the early stages of PD pathogenesis, so that therapies that target low-grade inflammation may be useful in treatment of PD.

In summary, we have found that extended doses of physiological concentrations of α-syn oligomers sensitizes the production of proinflammatory cytokines by the TLR4 receptor in both microglial cell lines and astrocytes and that the response increases with time. This observation provides an explanation for the switch to a proinflammatory response observed in PD and suggests, in combination with transcriptomic analysis, that sensitization of glial cells is a very early event in PD, driving the disease and increasing ROS production and cell death in neurons. Sensitization of the innate immune system by low concentrations of protein aggregates may also play an important early role in other neurodegenerative diseases. Rather than viewing PD as a protein aggregation disease, where increased protein aggregation initiates disease, our work suggests that PD can also be considered to be a neuroinflammatory disease, initiated by sensitized astrocytes and microglia.

## Electronic supplementary material

Below is the link to the electronic supplementary material.

Supplementary material 1 (DOCX 1364 kb)
