# Supplementary material for: Picomolar concentrations of oligomeric alpha-synuclein sensitizes TLR4 to play an initiating role in Parkinson’s disease pathogenesis
Source: Acta Neuropathol. 2018 Sep 17;137(1):103–20. doi: 10.1007/s00401-018-1907-y (PMC6338693; doi:10.1007/s00401-018-1907-y)
Supplement: Supplementary file 1 — Supplementary material 1 (DOCX 1364 kb) [file 401_2018_1907_MOESM1_ESM.docx]

**Supplementary Figures**


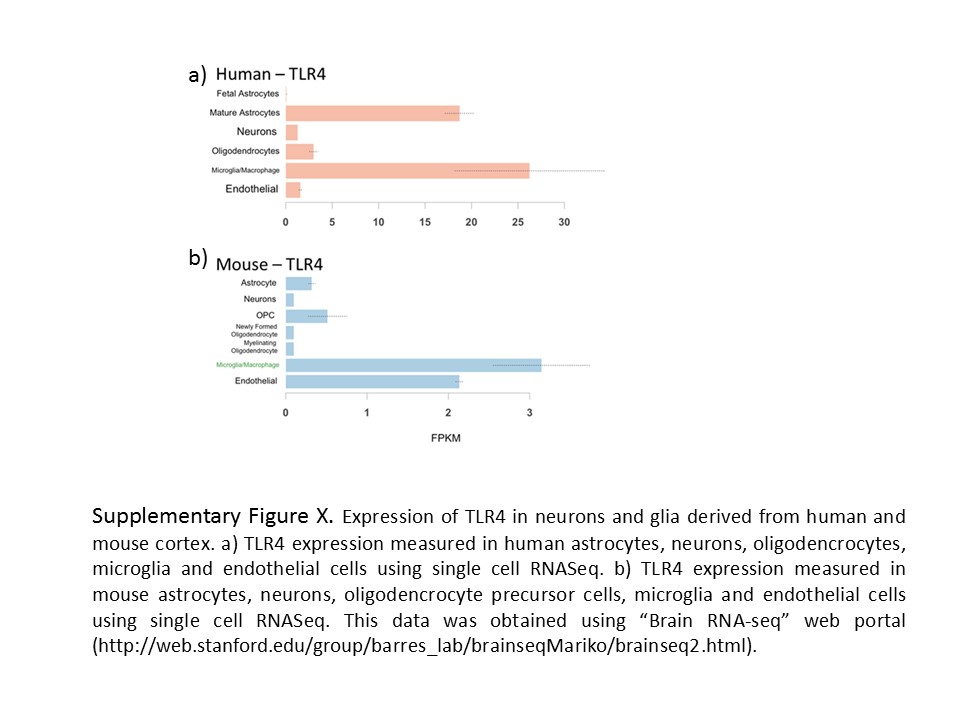


Supplementary Figure 1 , supporting Figure 1. Expression of TLR4 in neurons and glia derived from human and mouse cortex, obtained using “Brain RNA-seq”web portal

(<http://web.stanford.edu/group/barres_lab/brainseqMariko/brainseq2.html>.)

a) TLR4 expression measured in human astrocytes, neurons, oligodencrocytes, microglia and endothelial cells using single cell RNASeq. b) TLR4 expression measured in mouse astrocytes, neurons, oligodencrocyte precursor cells, microglia and endothelial cells using single cell RNASeq.


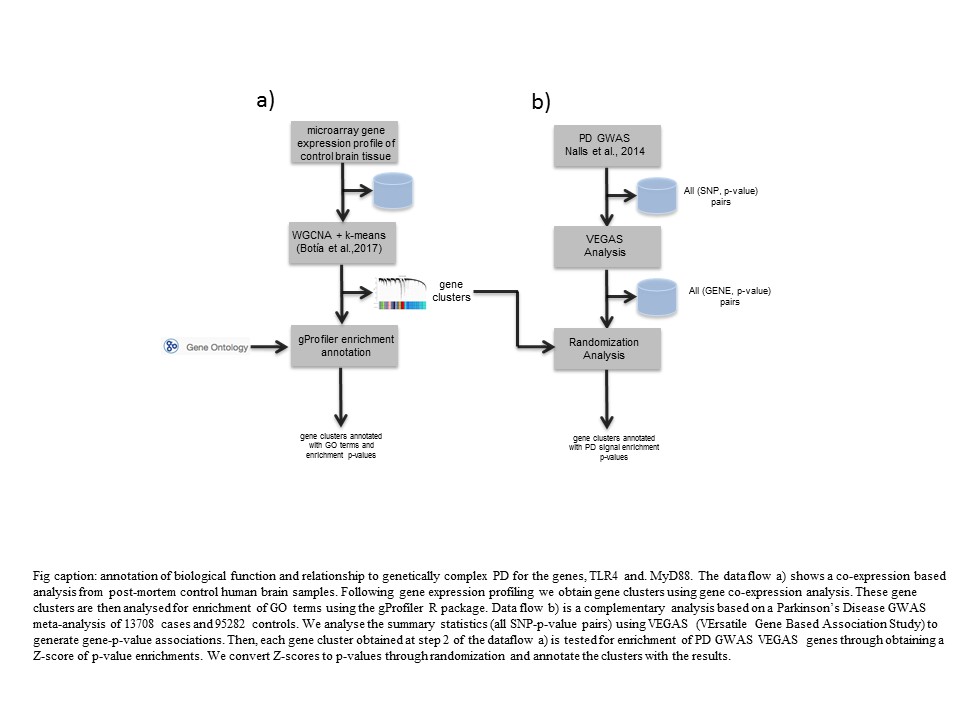


Supplementary Figure 2, supporting Figure 1: annotation of biological function and relationship to genetically complex PD for the genes, TLR4 and. MyD88. The data flow A) shows a co-expression based analysis from post-mortem control human brain samples. Following gene expression profiling we obtain gene clusters using gene co-expression analysis. These gene clusters are then analysed for enrichment of GO terms using the gProfiler R package. Data flow B) is a complementary analysis based on a Parkinson’s Disease GWAS meta-analysis of 13708 cases and 95282 controls. We analyse the summary statistics (all SNP-p-value pairs) using VEGAS (VErsatile Gene Based Association Study) to generate gene-p-value associations. Then, each gene cluster obtained at step 2 of the dataflow A) is tested for enrichment of PD GWAS VEGAS genes through obtaining a Z-score of p-value enrichments. We convert Z-scores to p-values through randomization and annotate the clusters with the results.


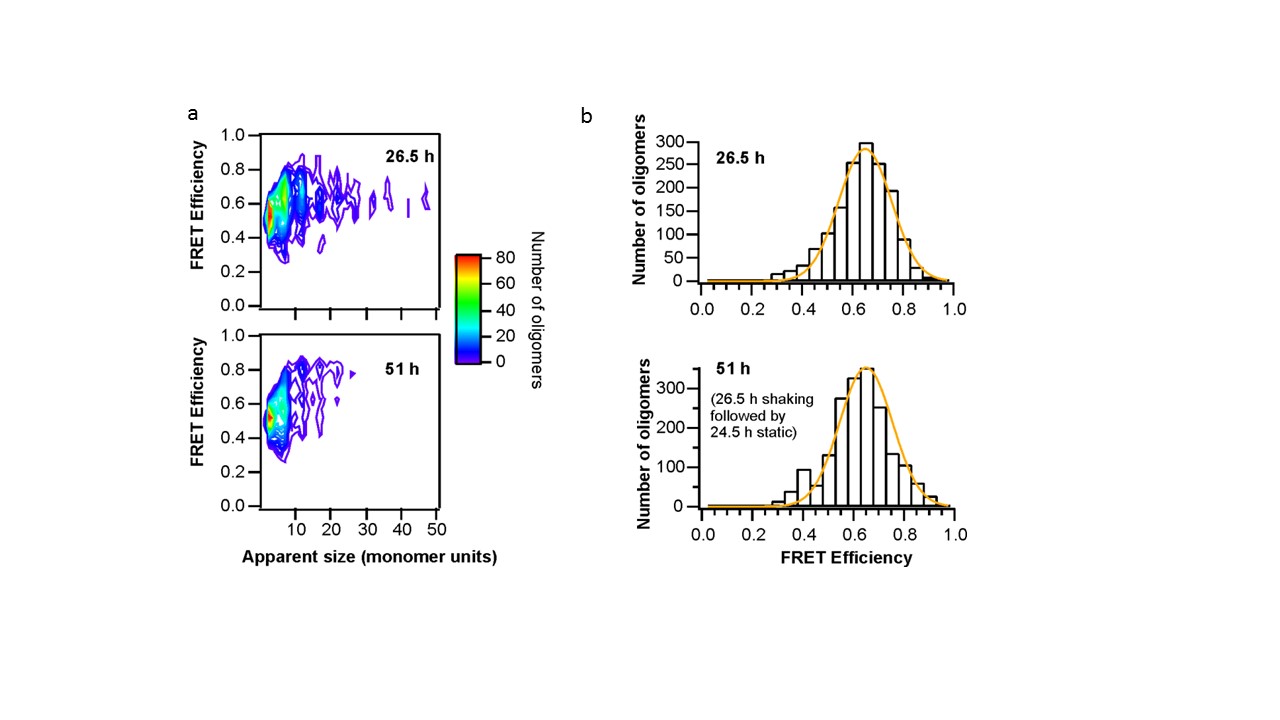


Supplementary Figure 3, supporting Figure 2. a) Two-dimensional contour plots of apparent size and FRET efficiency distributions of oligomers, formed in solutions of dye –labeled α-syn after 26.5 h of aggregation under shaking conditions in DMEM buffer. The solutions were analyzed immediately upon withdrawal (top), and following further 24.5-h incubation at 37 °C under quiescent conditions (bottom) to confirm oligomer stability during this incubation time. b) Corresponding FRET efficiency histograms of 6-150-mers, fitted to Gaussian distribution with average FRET efficiency, E=0.65, corresponding to high-FRET oligomer population.


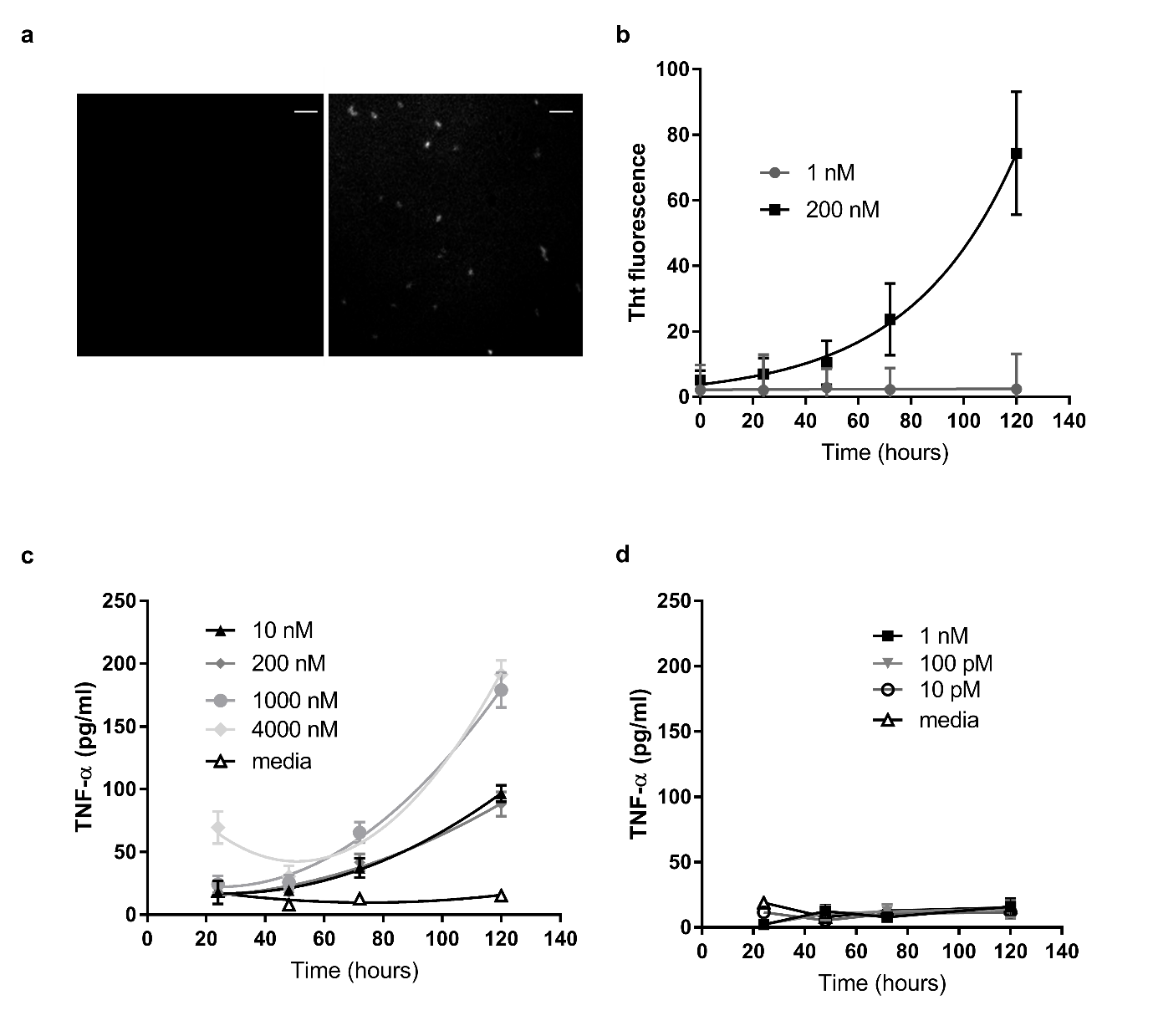


Supplementary Figure 4, supporting Figure 2. Formation of oligomeric α-syn from unlabeled monomers. a) TIRF image of the α-syn oligomers present in an initial solution of 200 nM monomer at 0 hours (left panel) and 120 hours (right panel) of incubation with BV2 microglia cells. Scale bar represents 5 µm. b) ThT assay of monomeric α-syn (1 nM and 200 nM) (n=3,sem). c) Pro-inflammatory response measured by the production of TNF-α in BV2 microglia after incubation of the cells with α-syn monomers (n = 3, sem). d) Pro-inflammatory response measured by the production of TNF-α in BV2 microglia after incubation of the cells with treatment of α-syn monomers, added at physiological concentrations (n =3, sem).


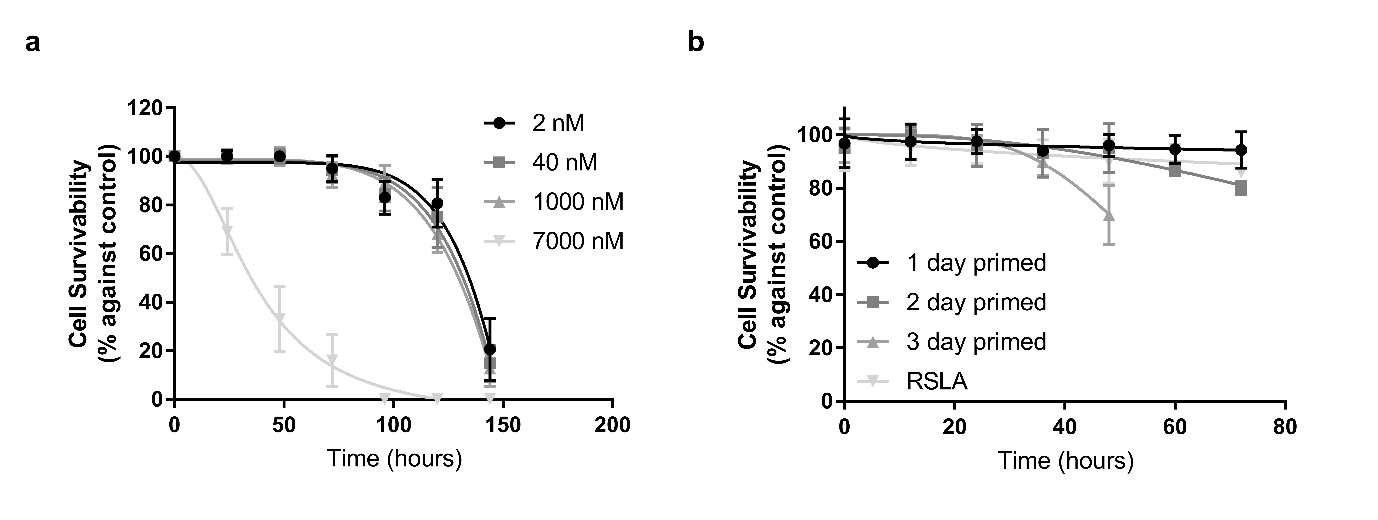


Supplementary Figure 5, supporting Figure 4. BV2 microglia cell survivability after incubation with different concentrations of α-syn oligomers. a) BV2 cells were incubated for 144 hours with oligomeric at a concentration of 2 nM, 40 nM, 1000 nM and 7000 nM with buffer exchanged every 24 hours. Cell counts were taken every 24 hours. Cell populations did not decrease below 88% (96-h, 1000 nM) of original unless under higher concentrations (n=3,p<0.00955, sem). b) BV2 cell were primed by incubating with 1000 nM oligomeric α-syn for 24, 48 or 72 hours. Cell counts were taken at 0-h, 24-h,48-h and 72-h after removal of oligomeric synuclein, followed by treatment with fresh oligomeric α-syn (1000 nM), with 100 ng/ml RSLA or 100 ng/ml RSLA only (n=3, sem).


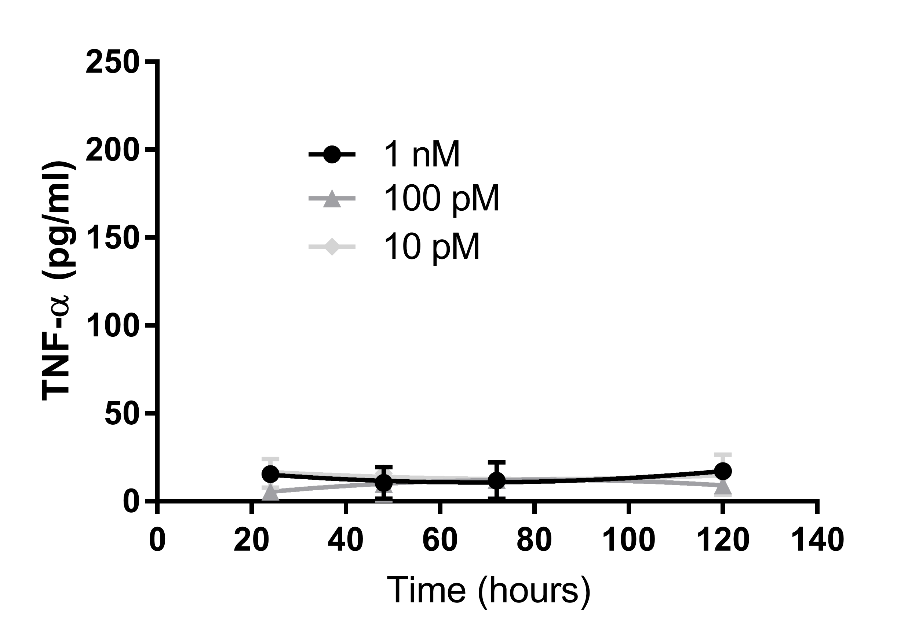


Supplementary Figure 6, supporting Figure 4. Pro-inflammatory response to different concentrations of α-syn oligomers measured in TLR4 knock out macrophages (n=3, SEM). TLR4 knock out cells produced negligible TNF-α levels over a 5-day period irrespective of the α-syn oligomer concentrations (p=0.135)


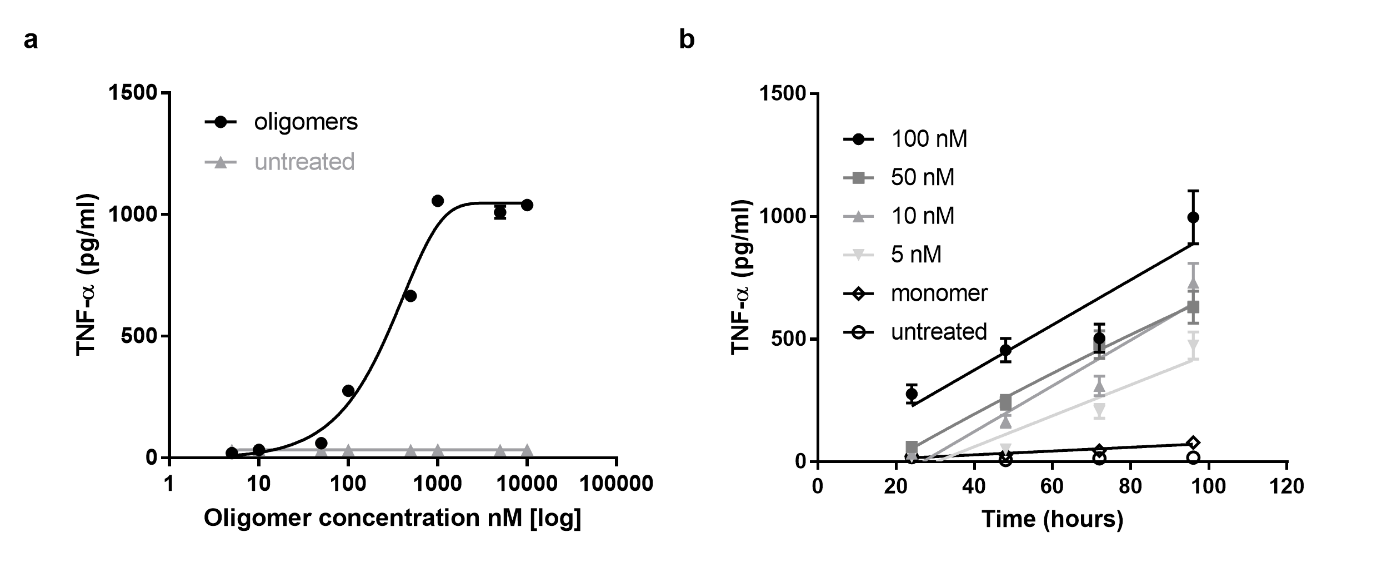


Supplementary Figure 7, supporting Figure 4. Pro-inflammatory response measured in human astrocytes after incubation with oligomeric α-synuclein. a) The pro-inflammatory response of human astrocytes measured by TNF-α production after a 24-h incubation with α-syn oligomers compared to untreated cells (p=0.00982) (n=3, sem). b) Time course of TNF-α production by human astrocyte cells in response to sustained exposure to αS-oligomers (5 nM-100 nM (n=3, sem)). Buffer was exchanged every 24 hours. No significant difference was observed between monomer and untreated data (p=0.0621) however a significant increase was observed between the untreated and 5 nM (p=0.0479), 10 nM (p=0.0365), 50 nM (p=0.0342) and 100 nM (p=0.0161). All statistical comparisons among groups were performed using one-way ANOVA with post hoc Tukey test.


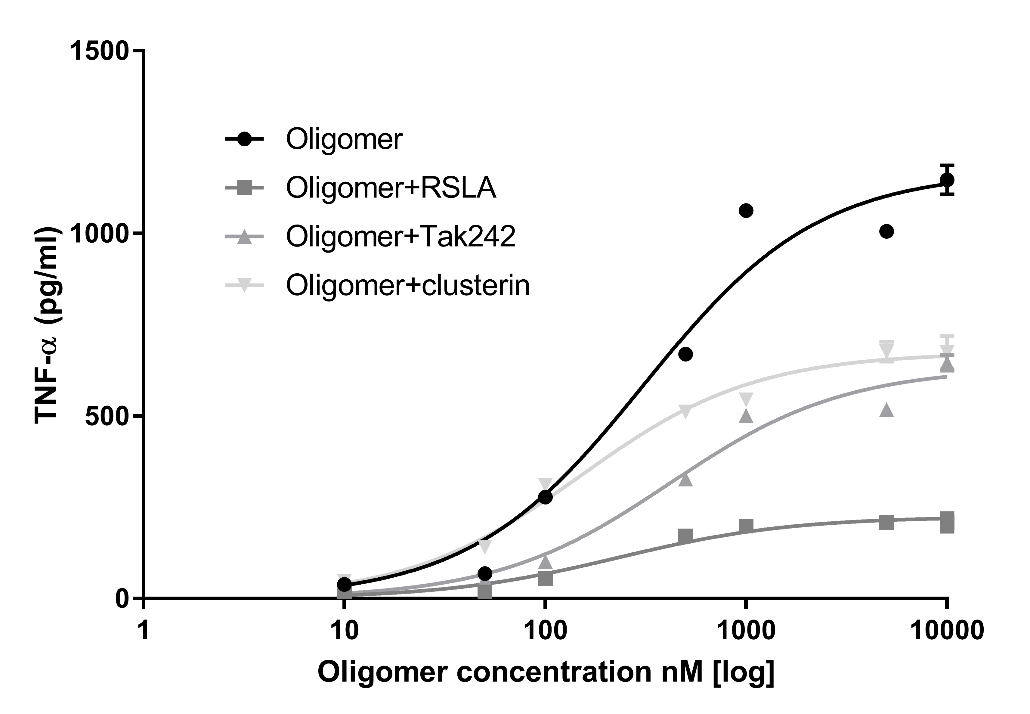


Supplementary Figure 8, supporting Figure 4. Pro-inflammatory response measured in human astrocytes after incubation with oligomeric α-syn and TLR4 antagonists and clusterin (n=4, sem). A significant decrease is observed between oligomer and RSLA (p=0.0087), oligomer and Tak242 (p=0.0222) and oligomer and clusterin (p=0.0387) (one way ANOVA with post hoc Tukey test).


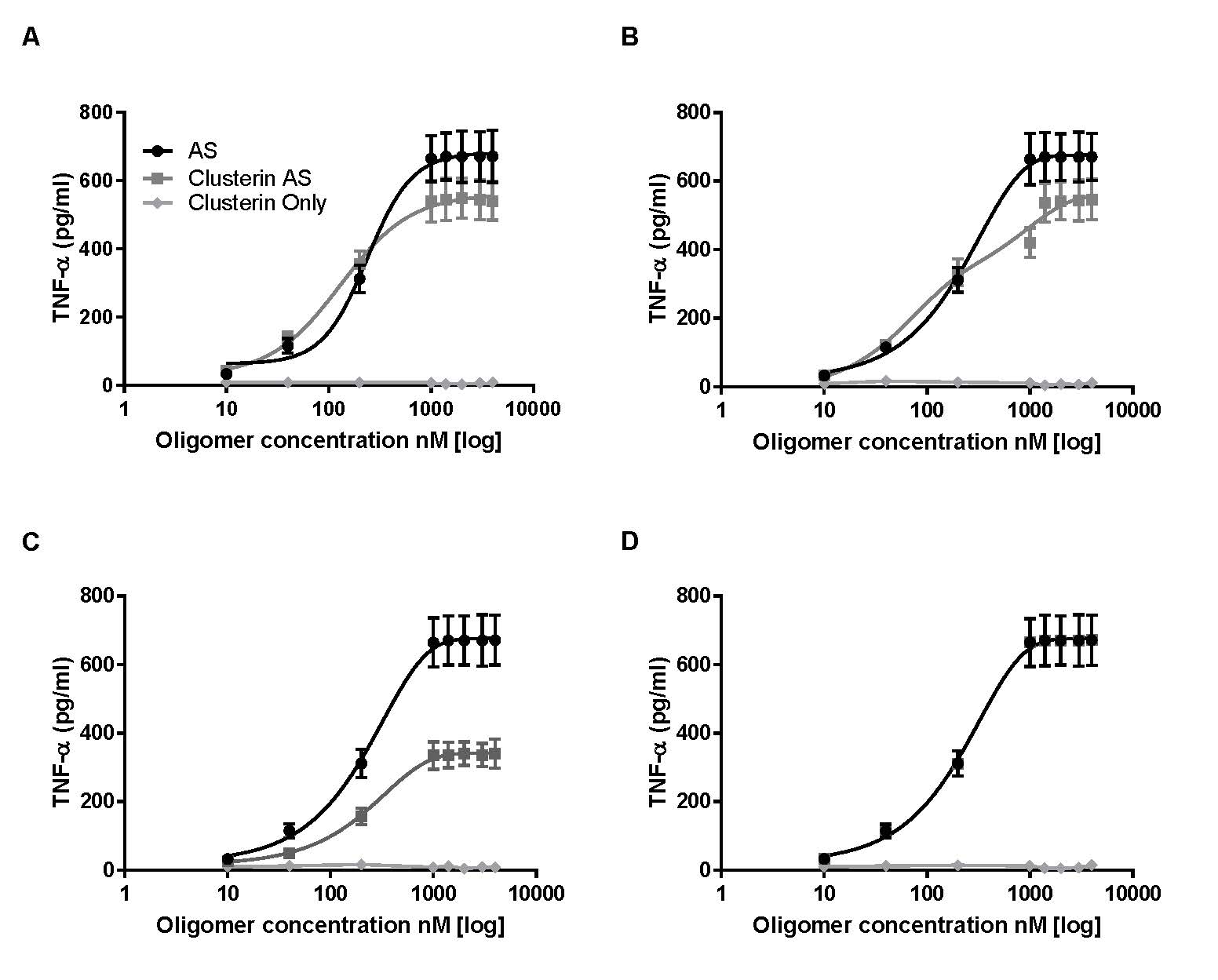


Supplementary Figure 9, supporting Figure 5. Pro-inflammatory response measured in BV2 microglia after 24-h incubation with oligomeric α-syn (1.5%) and monomer only (98.5%), oligomeric α-syn and monomer with clusterin and clusterin only (n=3, sem). a) The pro-inflammatory response when clusterin was added at the same concentration as the α-syn monomer (p=0.11 v oligomer only). b) The pro-inflammatory response when clusterin was 10 fold greater than the α-syn monomer (p=0.046 v oligomer only) c). The pro-inflammatory response when clusterin was 100 fold greater than the α-syn monomer (p=0.0088 v oligomer only). d) Pro-inflammatory response when clusterin concentrations were a 1000 times greater than that of the α-syn monomer (p=0.0011 v oligomer only). All statistical comparisons among groups were performed using one-way ANOVA with post hoc Tukey test.


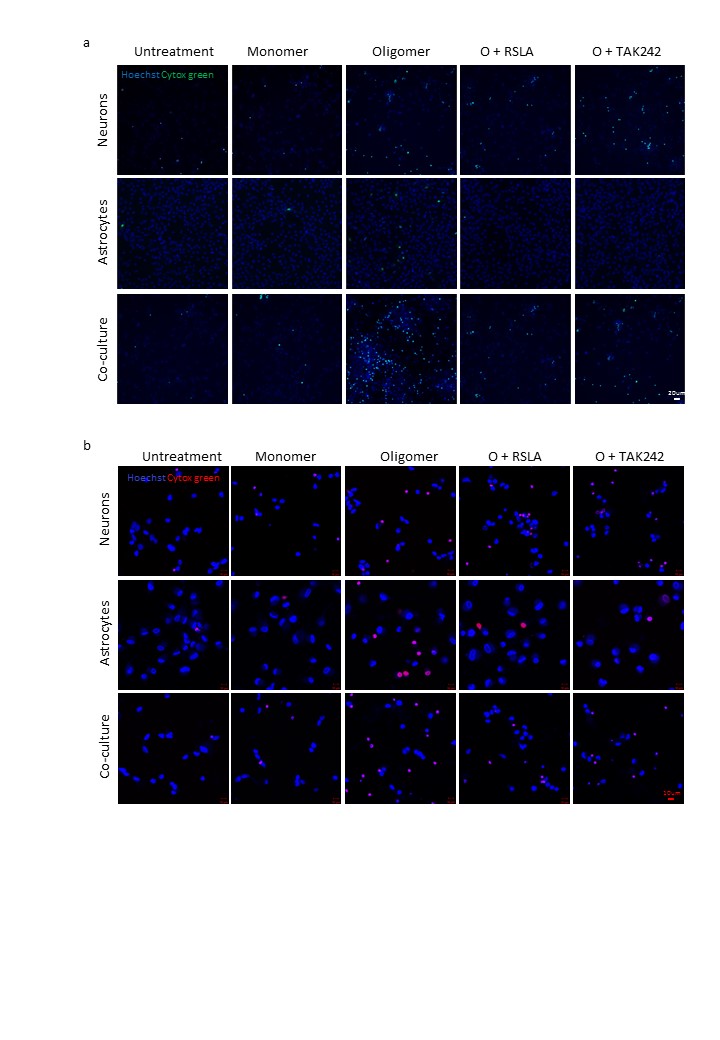


Supplementary Figure 10, supporting Figure 6. a & b) Representative images of the cell death experiment from Fig 6c & d. Images in (a) were obtained from rodent cells using a widefield microscope (high-throughput) and in (b) were from the human cell preparation imaged using a confocal microscopy. Scale bar = 10µm.


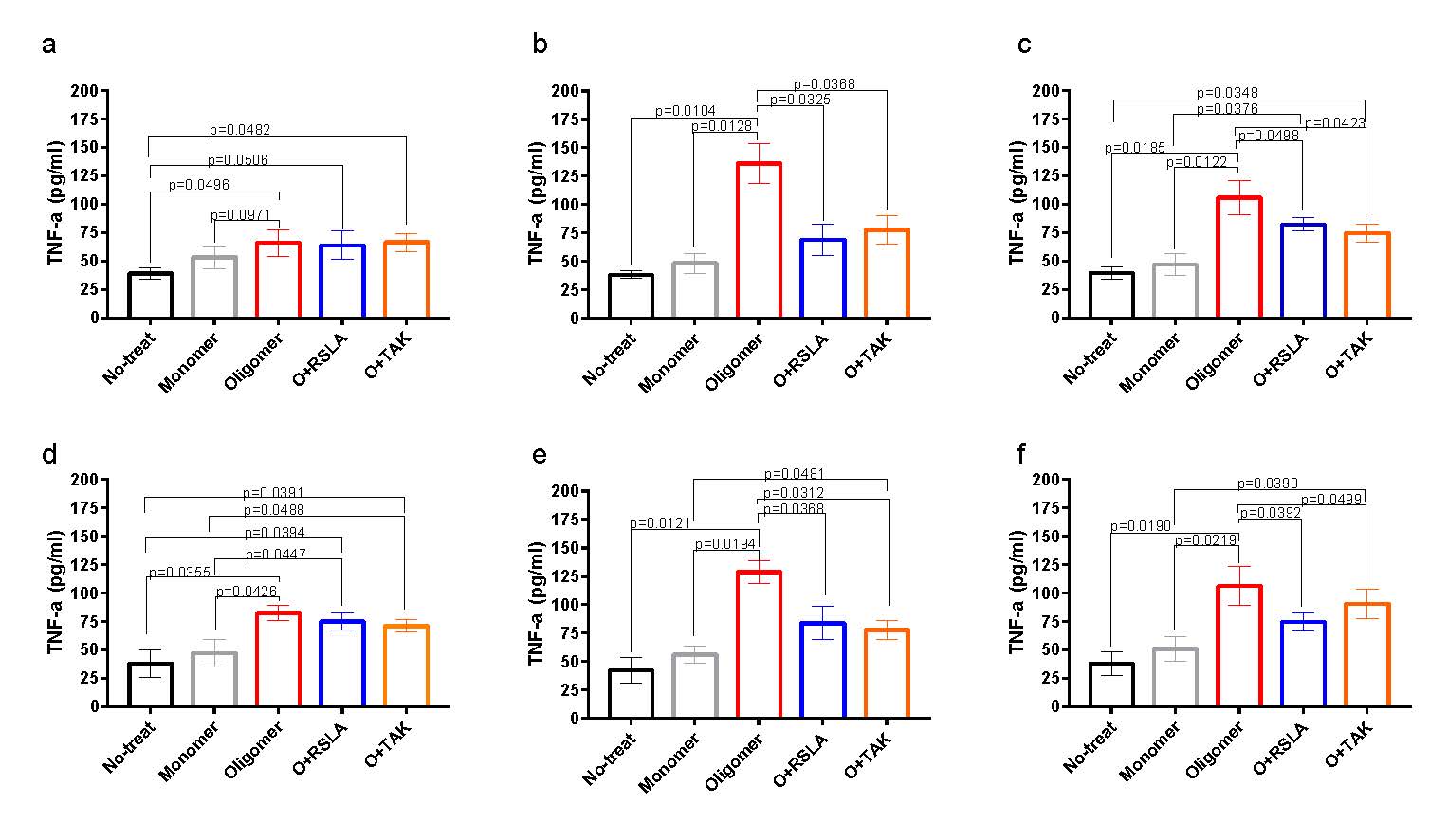


Supplementary Figure 11 supporting Figure 6. Oligomer induced cytokine response and dependence on TLR4. An oligomeric solution containing 1 µM monomer and 10 nM oligomer were  used with and without TLR4 inhibitors (RSLA and TAK242). a) to c) TNF-α response in enriched rodent neurons and astrocytes and co-cultures: a) neurons, b) astrocytes and c) co-cultures. d) to f) response in enriched human cells: d)neurons, e) astrocytes and f) co-cultures. (n=2, sem). No-treat: no treatment, Monomer: 1μM a-syn monomer, Oligomer; 10nM a-syn oligomer, O+ RSLA: 10nM a-syn oligomer + 0.1ug/ml RSLA, O+TAK: 10nM a-syn oligomer + 1μM TAK242. All statistical comparisons among groups were performed using one way ANOVA, followed by the post-hoc Tukey test.


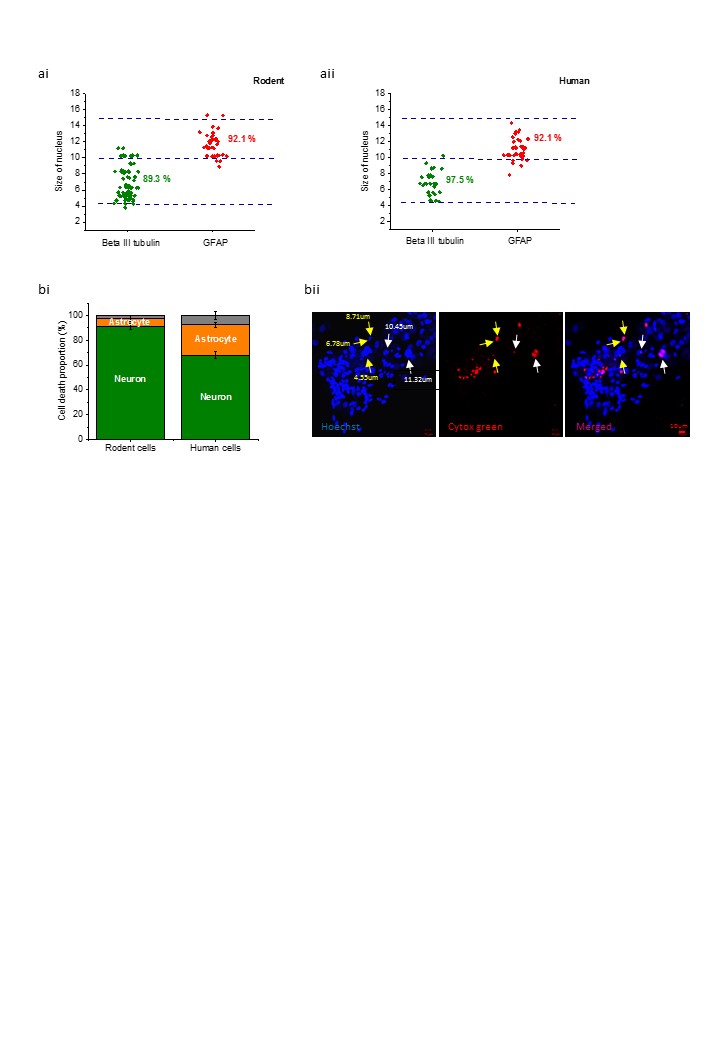


Supplementary Figure 12. Nuclear size were measured for neurons or astrocytes identified using the markers β-III Tubulin and GFAP in each enriched preparation. ai) In rodent cells, 89.3% of the β-III Tubulin +ve cells had nuclear size between 4.5µm and 10µm in the enriched neuronal culture (neuron prep) and 92.1% GFAP +ve cells had nuclear size between 10 µm and 15 µm in the enriched astroctye culture (AS prep). aii) For cells derived from human sources, 97.5% of neurons had nuclei sized between 4.5µm and 10µm in the neuron prep and 92.1% astrocytes had nuclei sized above 10µm in the AS prep. bi) In co-culture preparation, the proportion of neuronal and astrocytic death by oligomers was then analyzed based on the nuclear size (neurons: 4.5µm-10 µm; astrocytes 10 µm-15 µm, data collected from Figure6 cii & eii). In both rat and human cell preparations, the majority of oligomer induced cell death by oligomers was neuronal (91 ± 2.6 % and 68 ± 2.88 % of dead cells were neurons respectively). cii) Images showing examples of neurons (marked by yellow arrows) and astrocytes (marked by white arrows). Scale bar = 10µm.
